# Enabling selective zinc-ion intercalation by a eutectic electrolyte for practical anodeless zinc batteries

**DOI:** 10.1038/s41467-023-38460-2

**Published:** 2023-05-27

**Authors:** Chang Li, Ryan Kingsbury, Arashdeep Singh Thind, Abhinandan Shyamsunder, Timothy T. Fister, Robert F. Klie, Kristin A. Persson, Linda F. Nazar

**Affiliations:** 1 Department of Chemistry and the Waterloo Institute for Nanotechnology, University of Waterloo, Ontario, ON N2L 3G1 Canada; 2 Joint Center for Energy Storage Research, Argonne National Laboratory, Lemont, IL 60439 USA; 3 Energy Storage and Distributed Resources Division, Lawrence Berkeley National Laboratory, 1 Cyclotron Road, Berkeley, CA 94720 USA; 4 Department of Physics, University of Illinois - Chicago, Chicago, IL 60607 USA; 5 Chemical Sciences and Engineering Division, Argonne National Laboratory, Lemont, IL 60439 USA; 6 Molecular Foundry, Lawrence Berkeley National Laboratory, 1 Cyclotron Road, Berkeley, CA 94720 USA; 7 Department of Materials Science and Engineering, UC Berkeley, Berkeley, CA 94720 USA

**Keywords:** Batteries, Batteries, Batteries

## Abstract

Two major challenges hinder the advance of aqueous zinc metal batteries for sustainable stationary storage: (1) achieving predominant Zn-ion (de)intercalation at the oxide cathode by suppressing adventitious proton co-intercalation and dissolution, and (2) simultaneously overcoming Zn dendrite growth at the anode that triggers parasitic electrolyte reactions. Here, we reveal the competition between Zn^2+^ *vs* proton intercalation chemistry of a typical oxide cathode using ex-situ/*operando* techniques, and alleviate side reactions by developing a cost-effective and non-flammable hybrid eutectic electrolyte. A fully hydrated Zn^2+^ solvation structure facilitates fast charge transfer at the solid/electrolyte interface, enabling dendrite-free Zn plating/stripping with a remarkably high average coulombic efficiency of 99.8% at commercially relevant areal capacities of 4 mAh cm^−2^ and function up to 1600 h at 8 mAh cm^−2^. By concurrently stabilizing Zn redox at both electrodes, we achieve a new benchmark in Zn-ion battery performance of 4 mAh cm^−2^ anode-free cells that retain 85% capacity over 100 cycles at 25 °C. Using this eutectic-design electrolyte, Zn | |Iodine full cells are further realized with 86% capacity retention over 2500 cycles. The approach represents a new avenue for long-duration energy storage.

## Abstract (web summary)

Achieving high-performance aqueous Zn-metal batteries is a challenge. Here, authors report a eutectic electrolyte that concurrently enables selective Zn^2+^ intercalation at the cathode and highly reversible Zn metal plating/stripping, resulting in a benchmark high-areal capacity Zn anode-free cell.

## Introduction

Rechargeable aqueous zinc metal batteries (ZMBs) benefit from the use of zinc as the anode, owing to its high abundance, low cost, excellent compatibility with aqueous electrolytes, and high volumetric capacity (5850 Ah L^−1^)^1^. These merits have stimulated a surge of interest in exploring ZMBs that function in close-to-neutral aqueous media as a safe and low-cost electrochemical storage technology for sustainable large-scale stationary energy storage devices^2^. However, commercialization of ZMBs is still hindered by obstacles including (1) unfavorable proton intercalation and deleterious transition-metal dissolution of oxide cathodes at practical loadings and rates^3–5^; and (2) Zn dendrite formation and its parasitic reactions with the electrolyte that lead to unsatisfactory anode reversibility^6^.

Currently, limited studies have been devoted to the issues of oxide cathodes^7^. Proton co-intercalation is an intrinsic parasitic reaction occurring in these oxides during discharge in mildly-acidic dilute Zn electrolytes^3,4,8–10^, which favors high-rate cycling but compromises low-rate stability. Previous studies have demonstrated that layered double hydroxides (LDHs) always participate/dissolve on the surface of the cathode upon proton insertion/extraction, thus ensuring charge balance on the cathode/electrolyte interface^4,8^. The high mobility of protons in the hydrated lattice enables the high-rate capability of ZMBs, nonetheless, the facile detachment of LDHs from the surface, accompanied by cathode dissolution, causes considerable irreversibility. This is especially true at the low or moderate current densities required for long-duration energy storage (discharge on the order of 10 h^11^. The incorporation of protons in hydrated oxides can also induce structural failure and capacity decay upon cycling^9^. Lowering water activity using aqueous-nonaqueous electrolytes has been reported to potentially suppress proton intercalation^12,13^. For example, polyethylene glycol or propylene carbonate as crowding agents were reported to reduce water activity, hence lowering proton co-intercalation in phosphate-type cathodes, LiV_2_(PO_4_)_3_^12^ and VPO_4_F^13^, respectively. Enhanced Zn^2+^ intercalation was proven by Rietveld refinement of discharged materials. However, the efficacy of such approaches has not been verified in the more widely studied and high-capacity transition metal oxide cathodes. More serious concerns are the low (typically ~0.3 mAh cm^−2^) cathode loadings and ~0.2% Zn anode utilization due to poor Zn reversibility that were achieved in these studies, which are below the benchmark for translation and scale-up for practical ZMBs.

In contrast to limited efforts on the cathode, many approaches have been reported to enhance the reversibility and stability of the Zn anode. These include synergistic surface regulation of the Zn/substrate^14,15^, constructing an in/ex-situ solid-electrolyte interface (SEI)^16–20^, and utilizing three-dimensional porous zinc hosts^21–23^. Employing “water-in-salt” electrolytes (WiSEs) with reduced water activity also stabilizes the Zn anode^24,25^; however, the high cost of the salts or corrosivity compromises the economic benefits anticipated for ZMBs.

Recently, in the design of dilute-salt aqueous electrolytes, aqueous-nonaqueous hybrid solvents were reported for Li-ion batteries, where a portion of the water was substituted by organics, including polyethylene glycol (PEG)^26^ and sulfolane^27–29^. Similar concepts were then applied to ZMBs^12,30–37^. A hydrated eutectic electrolyte—incorporating succinonitrile as the solvent and hydrated Zn(ClO_4_)_2_ as the salt—decreases the water activity with improved Zn anode stability and suppressed cathode dissolution^30^. Using glycols^18,32^ or alcohols^31,38^ as the crowding agents also results in Zn plating/stripping with >99.5% coulombic efficiency (CE). A critical similarity among these studies is the displacement of water by nonaqueous solvents in the Zn^2+^ solvation structure, thus reducing the water activity. Unfortunately, a partially hydrated Zn^2+^ solvation sheath has a greater desolvation penalty than a fully hydrated one^39^, and that can result in sluggish interfacial charge transfer and high Zn deposition overpotentials. Moreover, most studies utilized low areal capacities of the cathode (<1 mAh cm^−2^), high C-rates (5–50 C), or impractically low Zn utilization (<1%), and did not address deleterious proton intercalation and cathode dissolution. Therefore, developing new approaches to suppress proton intercalation at a practical level is an ongoing challenge to realize ZMBs that can cycle at high-areal capacities (>3 mAh cm^−2^), moderate rates (<1 C), and high Zn utilization (>30%)^5^.

Herein we elucidate the Zn^2+^ vs. proton intercalation mechanism of a metal oxide cathode (Zn_0.25_V_2_O_5_·*n*H_2_O (ZVO)) in various electrolytes by a combination of ex-situ/*operando* techniques. A strategy of employing a sulfolane-based hybrid eutectic electrolyte effectively suppresses deleterious proton intercalation and cathode dissolution. This is ascribed to a strongly hydrogen-bonded network of sulfolane-water that alleviates water activity. A fully hydrated Zn^2+^ solvation structure is revealed and facilitates interfacial charge transfer. Highly stable and reversible Zn plating/stripping is demonstrated in both Zn||Cu and Zn||Zn cells. As a consequence of stabilizing both electrodes in the hybrid eutectic electrolyte, we report the first practical high-areal-capacity (4 mAh cm^−2^) Zn anode-free cell that can run over 100 cycles at 0.15 C with ~ 85% capacity retention.

## Results

### Electrolyte network of hybrid eutectic electrolytes

**Fig. 1:**
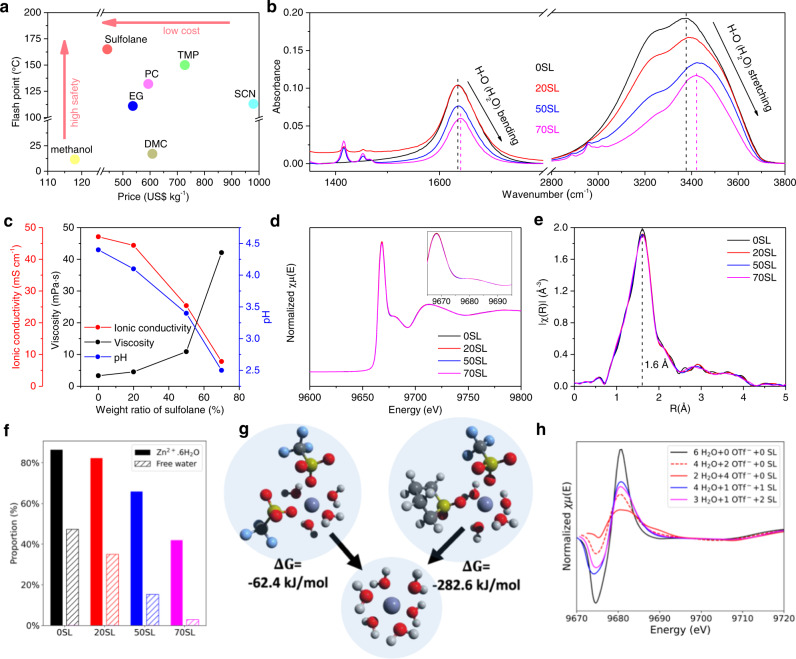
Electrolyte network of hybrid eutectic electrolytes. **a** Price and flash points of various nonaqueous solvents: methanol, sulfolane, ethylene glycol (EG), propylene carbonate (PC), dimethyl carbonate (DMC), trimethyl phosphate (TMP), and succinonitrile (SCN). Details can be seen in Supplementary Table 1. **b** FTIR spectral evolution of different electrolytes. **c** Evolution of ionic conductivity, viscosity, and pH at various weight ratios of sulfolane. **d**, **e** XANES (**d**) and EXAFS (**e**) spectra of different electrolytes. **f** Proportion of fully hydrated Zn^2+^ (filled bars) and free water (hatched bars) from classical MD simulation of bulk electrolytes. **g** DFT-computed reaction energies to form fully hydrated Zn^2+^ from other cluster types (Zn^2+^·4H_2_O·2OTf^-^, left, or Zn^2+^·4H_2_O·1OTf^-^·1SL, right). **h** Simulated XANES spectra associated with different solvated cluster types.

We chose sulfolane (SL) as the crowding agent because of its low-cost; high flash and boiling points (Fig. 1a and Supplementary Table 1); and ability to act as a second hydrogen bond acceptor to reduce water activity^34,36^. Zn(OTf)_2_ exhibits high solubility (Supplementary Fig. 1), and reduced desolvation penalty owing to the bulky OTf^-^ anion; good compatibility with Zn^40–42^, and relatively low cost compared to other bulky-anion salts^18^. Through experimental trials, we find that 7:3 (denoted as 70SL, see Methods), weight/weight, is the highest ratio of SL/water that can form the eutectic electrolyte with a low melting point of −81.6 °C (Supplementary Fig. 2) and adequate solubility of salts (2 m, molality) at 25 °C. Flammability tests show the hybrid electrolyte cannot be ignited with a flame (Supplementary Movies 1, 2), suggesting excellent potential as a safe and cost-effective electrolyte for sustainable large-scale energy storage.

The reduced water activity of 70SL relative to 0SL was demonstrated by the blue shift of both bending and stretching modes of the O-H bond of H_2_O in Fourier Transform Infrared spectra (FTIR, Fig. 1b), suggesting a strengthened O-H bond due to the strong interaction between SL and H_2_O. This is verified by the narrowing of the corresponding ^1^H peak in the ^1^H-NMR spectrum with the addition of SL, and 100% water retention of 70SL in an open atmosphere (Supplementary Fig. 3). While increased SL/water ratios give lower ionic conductivity (σ_i_) and higher viscosity (Fig. 1c), σ_i_ is sufficiently high (7.8 mS cm^−1^) for 70SL.

Intriguingly, the Zn^2+^ solvation structure is not altered by SL, as shown by the exactly overlapped X-ray absorption near-edge structure (XANES) spectra (Fig. 1d). Extended X-ray absorption fine structure (EXAFS) data (Fig. 1e) reveal a peak at 1.60 Å, exactly corresponding to the Zn-O bond length in Zn^2+^(H_2_O)_6_^43^. This was further confirmed by classical molecular dynamics (MD) simulations that show fully hydrated Zn^2+^ is the predominant solvated cluster type in 0SL, 20SL, and 50SL. Solvation shells containing triflate and/or SL become more prevalent as the SL loading increases (Fig. 1f), although Zn^2+^ was six-coordinate in all cases. The free water content also decreases with SL loading, in agreement with the increased water retention observed experimentally (Supplementary Fig. 3b). Although the MD simulations suggest that a significant fraction of Zn^2+^ clusters in 70SL are not fully hydrated, this result should be interpreted cautiously because competition between water, triflate, and SL in Zn^2+^ solvation was found to be particularly sensitive to the charge scaling protocol employed (see Methods). This reflects the inherent limitations of empirical, non-polarizable water models^44,45^. Indeed, first-principles reaction energies computed from density functional theory (DFT) indicate that Zn^2+^ clusters containing triflate or SL in their primary solvation shell will spontaneously revert to fully hydrated Zn^2+^ (Fig. 1g). Moreover, simulated XANES spectra of specific cluster types indicates that entry of triflate or SL into the primary Zn^2+^ solvation shell would result in features to the right of the primary *K*-edge peak at ~9685 eV (Fig. 1h), which were not observed in the experimental spectra (Fig. 1d). Taken together, the results support the view that a high proportion of fully hydrated Zn^2+^ is maintained at all SL content. Thus, facile interfacial charge transfer and a low Zn deposition overpotential can be anticipated owing to the low desolvation penalty of the (Zn(H_2_O)_6_)^2+^ cation^39^. It should be noted that partially hydrated solvation structures based only on MD simulations were proposed in previously reported hybrid electrolytes^12,30–37^. Nonetheless, such simulation results should be cautiously interpreted due to the complicated parameter optimization as mentioned above. We believe that a combination of conclusive experimental analysis (for example, XAS spectra), MD simulations and DFT calculations are necessary to identify the Zn^2+^ solvation sheath in hybrid electrolytes.

### Zn^2+^ vs. proton intercalation electrochemistry in the metal oxide cathode

**Fig. 2:**
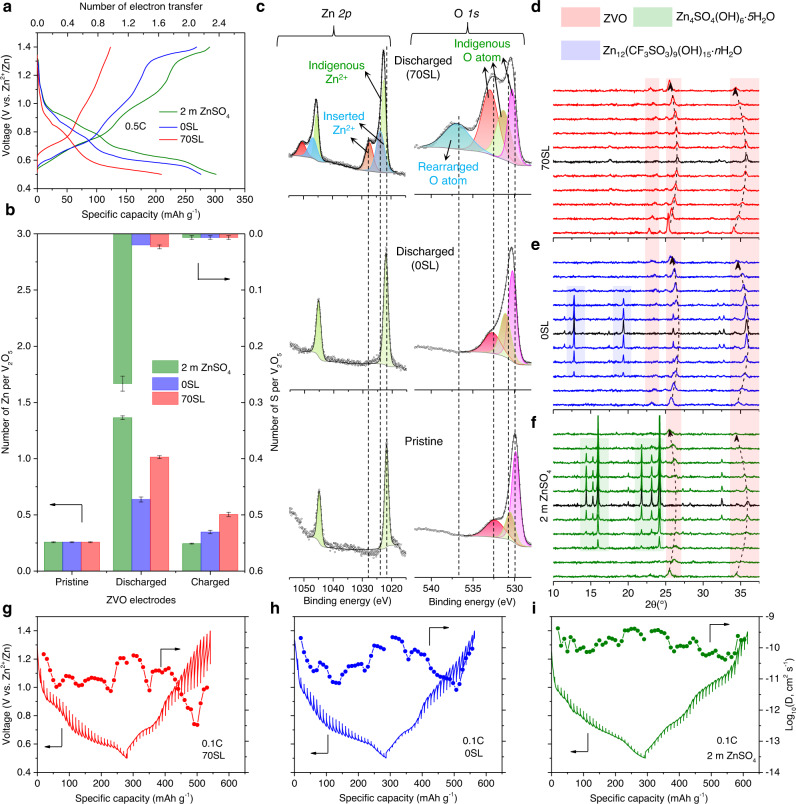
Zn^2+^ vs. proton intercalation electrochemistry of ZVO cathode. **a**, **b** Voltage profiles of ZVO during the first cycle (**a**) and corresponding EDS-derived elemental ratio of Zn, V, and S at different electrochemical states (**b**). The error bars are derived from the mathematical average of the EDX ratios in three different areas of each electrode. **c** Zn 2*p* and O 1*s* XPS spectra of pristine ZVO and discharged ZVO in 0SL and 70SL. **d**–**f** *Operando* XRD patterns of ZVO in 70SL (**d**), 0SL (**e**), and 2 m ZnSO_4_ (**f**). **g**–**i** GITT voltage profiles of ZVO and corresponding average diffusion coefficients in 70SL (**g**), 0SL (**h**), and 2 m ZnSO_4_ (**i**).

Zn_0.25_V_2_O_5_·nH_2_O (ZVO) was selected as a representative cathode to investigate Zn^2+^ vs. proton intercalation in various electrolytes. Figure 2a shows the initial voltage profiles of ZVO | |Zn full cells at 0.5 C (1 C = 300 mA g_zvo_^−1^) in three electrolytes: 2 m ZnSO_4_ in water; 2 m Zn(OTf)_2_ in water (0SL), and 2 m Zn(OTf)_2_ in 7:3 sulfolane/water (70SL), where the discharge capacities are 302 mAh g_zvo_^−1^ (2.3 electron (*e*^−^)), 276 mAh g_zvo_^−1^ (2.2 *e*^*−*^), and 210 mAh g_zvo_^−1^ (1.6 *e*^*−*^), respectively. In 70SL, a higher cell polarization is observed with 60% discharge capacity recovered on subsequent charge; lower than the 97% capacity recuperated in either 2 m ZnSO_4_/water or 0SL in the voltage window 1.4 - 0.5 V vs Zn^2+^/Zn. Energy-dispersive X-ray spectroscopy (EDS) analysis reveals ~1.01 Zn^2+^ per formula unit in ZVO discharged in 70SL (Fig. 2b and Supplementary Figs. S4–S7), i.e., a composition of Zn_1.01_V_2_O_5_, and intercalation of ~0.76 Zn^2+^ (after accounting for ~0.25 indigenous Zn^2+^). This accounts for 95% of the initial discharge capacity, as opposed to only 35% in 0SL (~0.38 Zn^2+^ intercalated, based on EDS analysis). In 2 m ZnSO_4_, the discharged sample shows high sulfur content (Fig. 2b), originating from sulfate-based LDH (Zn_4_(SO_4_)(OH)_6_·*5*H_2_O, Supplementary Fig. 8). Washing discharged electrodes with dilute acid has been used to dissolve LDHs from the surface in order to evaluate the quantity of intercalated Zn^2+^ ^3,4^; nonetheless, we now believe that rapid ion exchange between Zn^2+^ and protons compromises the accuracy of this method (Supplementary Figs. 9–11 and Supplementary Table 2). For the charged sample in 70SL, the apparent limit of ~0.47 Zn^2+^ deintercalation matches well with the ~60% capacity recovery (Fig. 2b). This suggests that Zn^2+^ diffusion in ZVO is sluggish in the solid state, as verified by subjecting cells to a potential hold at the top of charge during cycling which greatly increased the subsequent discharge capacity (Supplementary Fig. 12).

XPS analysis provides further proof for dominant Zn^2+^ intercalation in 70SL (Fig. 2c). Compared to pristine ZVO, in the Zn *2p* region, two additional peaks appear at higher binding energies (1023.7 and 1027.4 eV) for the sample discharged in 70SL, which can be ascribed to the inserted Zn^2+^. However, in 0SL, only the peak (1022.1 eV) corresponding to the indigenous Zn^2+^ is observed with a slight blue shift. In the O 1*s* region of ZVO discharged in 70SL, a new distinct broad peak at 537.1 eV may indicate the concomitant bonding rearrangements of the oxygen atoms owing to the inserted Zn^2+^, which also induces a significant blue shift of the three peaks between 530–534 eV relative to that of pristine ZVO. In contrast, ZVO discharged in 0SL only shows a slight blue shift of the O 1*s* peaks, indicating that the inserted protons have an insignificant impact on the lattice oxide. This can be ascribed to the monovalent proton and its small size, whereas the larger divalent Zn^2+^ may distort the structure *via* strong interaction with the ZVO framework, thus altering the local oxide environment.

*Operando* XRD measurements were further conducted to elucidate structural differences upon intercalation of Zn^2+^ vs. H^+^. In 70SL (Fig. 2d), the progressive structural evolution of ZVO can be clearly identified by the reflections shifting to higher 2θ angles (lower d-spacing). The gradual lattice shrinkage during discharge is ascribed to the expulsion of interlayer water molecules upon Zn^2+^ insertion^46^ (Supplementary Fig. 13). In 0SL (Fig. 2e) and 2 m ZnSO_4_ (Fig. 2f), a set of strong reflections corresponding to triflate and sulfate-based LDHs appear, respectively^3,4^. These result from the local alkaline environment on the cathode interface because of dominant proton intercalation on discharge. The complete lack of LDH reflections in 70SL indicates alleviated proton intercalation and dominant Zn^2+^ intercalation. The charge process follows the reverse evolution of discharge in all three electrolytes, showing good structural reversibility of ZVO. In addition to majority proton intercalation in 0SL, significant cathode dissolution is also identified by the yellow color of the separator disassembled from cycled cells, in contrast to no apparent dissolution in 70SL (Supplementary Fig. 14).

To estimate Zn^2+^-ion diffusion kinetics in ZVO, we conducted galvanostatic intermittent titration technique (GITT) studies. ZVO in 70SL shows a relatively low average Zn^2+^-ion diffusion coefficient (D_eff_) ranging from 10^−10^–5 × 10^−12^ cm^2^ s^−1^ at most states of discharge/charge (Fig. 2g). A very low diffusion coefficient of ~5 × 10^−13^ cm^2^ s^−1^ was observed at the mid-point of charge at 1.2 V, perhaps explaining the low-capacity recovery observed during the first charge. ZVO cycled in 0SL and 2 M ZnSO_4_, however, shows more than one magnitude higher average D_eff_ (Fig. 2h, i), ranging from 5 × 10^−10^–10^−11^ and 10^−9^–5 × 10^−11^ cm^2^ s^−1^, respectively. These results support the substantial co-proton intercalation of ZVO in 0SL and 2 m ZnSO_4_, as H^+^ diffusion is expected to be much faster than that of Zn^2+^ions.

The above discussion highlights that the extremely high-rate function of ZMBs based on the oxides utilized in this work or elsewhere is likely only attainable with proton co-intercalation. Such batteries could potentially be used for high-power load leveling, but would be unable to support long-duration electricity energy storage that requires stable cycling at moderate current densities with discharge periods of nearly 10 h (see below)^11^.

### Evidence of Zn^2+^ intercalation from electron microscopy

**Fig. 3:**
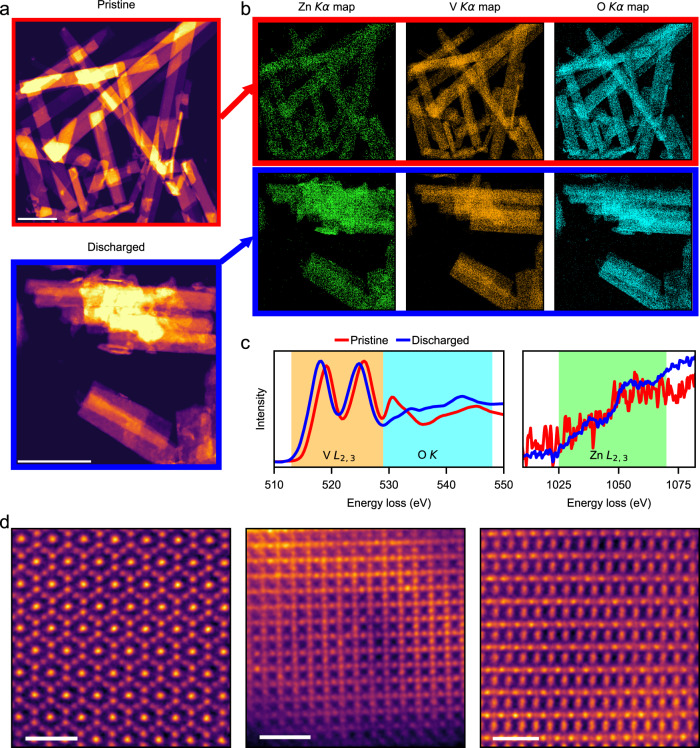
Proof of Zn^2+^ intercalation in 70SL from electron microscopy. **a** STEM-HAADF images showing the nanobelt-like morphology for the pristine (top) and discharged (bottom) ZVO. **b** EDS chemical maps showing the distribution of Zn, V, and O across the nanobelts in the pristine and discharged ZVO samples. **c** Integrated EEL spectra showing the changes in the fine structure of V *L*, O *K*, and Zn *L* edges for the pristine and discharged ZVO samples. **d** Atomic-resolution HAADF images showing various crystal structure orientations for the discharged ZVO samples. The scale bars correspond to 500 and 1 nm for (**a**) and (**d**), respectively.

Scanning transmission electron microscopy (STEM) imaging combined with energy-dispersive X-ray spectroscopy (EDS) and electron energy loss spectroscopy (EELS) was used to investigate the evolution of morphology, chemical distribution, electronic and crystal structures of the ZVO upon discharge in 70SL. Compared to the clean surface of pristine ZVO nanobelts in the high-angle annular dark-field (HAADF) image, the discharged sample exhibits corrosion-induced surface roughness typical of electrochemically cycled materials (Fig. 3a). The EDS chemical maps show homogeneously distributed Zn, V and O elements for both pristine and discharged nanobelts (Fig. 3b), with V/Zn ratios of 9.6 and 2.3, respectively. This agrees well with the intercalation of 0.7 Zn^2+^ per formula unit described above (Fig. 2b).

To understand the impact of Zn^2+^ intercalation on the electronic structure of ZVO, we show a comparison of the integrated EELS spectra (a total of ten spectra each) for the pristine and discharged ZVO samples in Fig. 3c. The reduction in the V valence state upon Zn^2+^ intercalation results in a shift of the V *L* edge towards lower energy loss^47,48^. The pre-peak in the O *K* edge is the result of electronic transitions from O 1*s* states to O 2*p* states. The O 2*p* states are hybridized with the V 3*d* states, and as a result, the higher O *K* edge pre-peak intensity points towards a higher number of empty O 2*p* states hybridized with V 3*d* states^49,50^. Therefore, the reduced intensity of the O *K* edge pre-peak is a direct result of V reduction upon Zn^2+^ intercalation. While the Zn *L* edge is extremely noisy for the pristine ZVO sample, the discharged sample shows a Zn *L* edge with a much higher signal-to-noise ratio due to the increased Zn content upon intercalation. Figure 3d shows atomic-resolution HAADF images corresponding to the various crystal structure orientations in the discharged ZVO samples. The atomically resolved HAADF images for pristine ZVO samples are shown in Supplementary Fig. 15.

### Highly reversible Zn plating/stripping and solid-electrolyte interface (SEI) chemistry

**Fig. 4:**
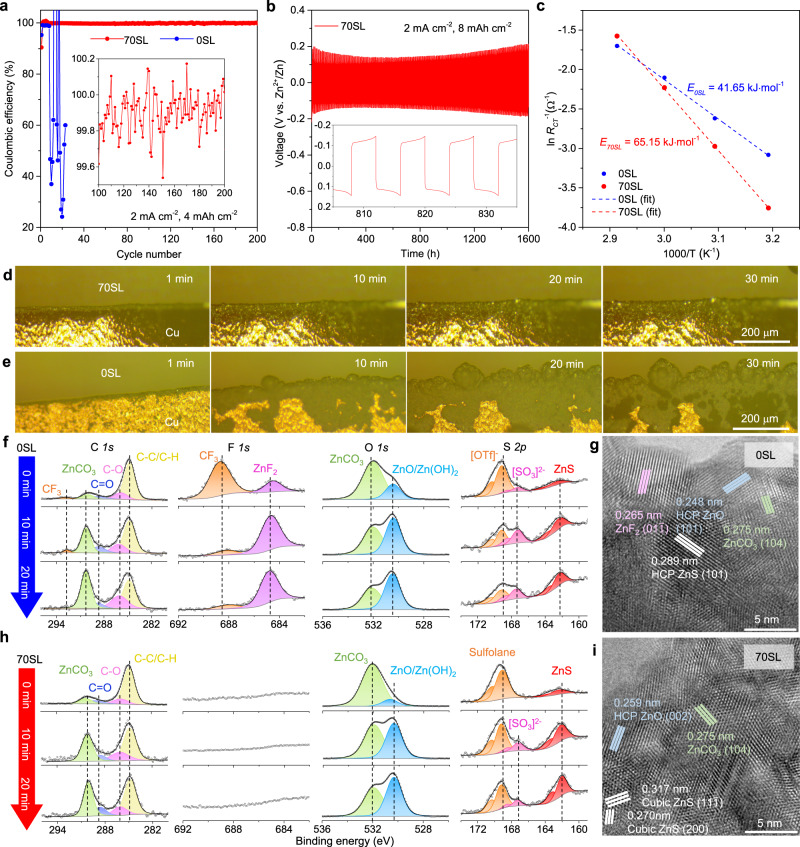
Dendrite-free and highly reversible Zn plating/stripping. **a** Coulombic efficiency of Zn||Cu cells at 2 mA cm^−2^ with a capacity of 4 mAh cm^−2^. **b** Galvanostatic Zn stripping/plating in Zn||Zn symmetric cells with 70SL at 2 mA cm^−2^ with a capacity of 8 mAh cm^−2^. **c** Arrhenius plots of inverse R_CT_ (R_CT_^−1^) values at different temperatures (from 40 to 70 °C) for the working electrode in a three-electrode cell with Zn as working, counter, and reference electrode; **d**, **e** In In-situ optical microscopy images showing the Zn plating process in 70SL (**d**) and 0SL (**e**) at 5 mA cm^−2^. **f**, **h** High-resolution depth-profiling XPS spectra of Cu electrodes with deposited Zn after 5 cycles at 2 mA cm^−2^ (4 mAh cm^−2^) in 0SL (**f**) and 70SL (**h**). **g**, **i** TEM images of the interfaces on the deposited Zn surface in 0SL (**g**) and 70SL (**i**).

Practical ZMBs also require anodes with high reversibility and stability. Zn||Cu asymmetric cells evaluated in 70SL demonstrated a very high Zn plating/stripping CE of at least 99% at different current densities (Supplementary Fig. 16a), which is slightly higher than that in 0SL (Supplementary Fig. 16b). The long-term Zn plating/stripping behavior was further evaluated at 2 mA cm^−2^. In Zn||Cu cells with a high-areal capacity of 4 mAh cm^−2^, the CE in 70SL (initially 90.4%) stabilizes at ~ 99.9% for 200 cycles (Fig. 4a and Supplementary Fig. 17), yielding a very high average CE of 99.8% without the presence of “soft shorts” (also see full-cell cycling below)^51^. In 0SL, in contrast, a large CE fluctuation owes to extensive dendrite formation. We also note 70SL shows significantly better results than 50SL in our study (Supplementary Fig. 18) or previous reports^35^, which can be ascribed to the lower free water content of ~3% in 70SL vs. ~16% in 50SL (Fig. 1f). This proves the critical role of SL/water ratio in these hybrid electrolytes. The excellent long-term stability of multiple Zn||Zn symmetric cells in 70SL (Fig. 4b and Supplementary Fig. 19) is demonstrated by a cycle life of at least 2000 h at 4 mAh cm^−2^, 1600 h at 8 mAh cm^−2^ and 1000 h at 12 mAh cm^−2^. The lifetime is extended 20-fold compared to cells employing 0SL at 8 mAh cm^−2^, which quickly short after only 80 h (Supplementary Fig. 20). The superior Zn anode stability in 70SL is further verified by very little change in charge transfer resistance (*R*_CT_) during the Zn plating/stripping (Supplementary Fig. 21).

The activation energy (*E*_a_) for the charge transfer process (Zn(H_2_O)_6_)^2+^↔ Zn^2+^↔ Zn^0^ on the Zn surface was measured by electrochemical impedance spectroscopy (EIS). As shown in Fig. 4c and Supplementary Fig. 22, the *E*_a_ of 70SL is 65.15 kJ mol^−1^, higher than the 41.65 kJ mol^−1^ for 0SL. The enthalpy (Δ*H*) and entropy (Δ*S*) of this interfacial charge transfer were also estimated using the Eyring–Polanyi equation (Supplementary Fig. 23). As 0SL and 70SL present similar fully hydrated Zn^2+^ solvation structures and presumably comparable desolvation energy, we ascribe the higher *E*_a_ and *R*_*CT*_ in 70SL to the slower diffusion of desolvated Zn^2+^ in the proposed sulfolane-derived SEI (see below) on the Zn surface and a slightly lower transference number (Supplementary Fig. 24). We note that the *E*_a_ of 70SL is still similar to that of dilute 2 M ZnSO_4_ (68.2 kJ mol^−1^)^52^, and more than three-fold lower than reported for 0.25 M Zn(OTf)_2_ in acetonitrile (~223.64 kJ mol^−1^)^39^, where a water-free Zn^2+^ solvation sheath and strong Zn^2+^-OTf^-^ ion pairing induce a much higher desolvation penalty. In contrast, fully hydrated (Zn(H_2_O)_6_)^2+^ in 70SL facilitates facile interfacial desolvation owing to the shielding effect of water^39^.

The superior Zn stability and reversibility exhibited in 70SL is attributed to suppressed HER kinetics (Supplementary Fig. 25) and improved compatibility with Zn (Supplementary Fig. 26). The lowered HER potential is ascribed to an in situ surface SEI (see below), which prevents water from approaching the interface but allows Zn^2+^ to pass through, and suppresses the formation of Zn layered double hydroxides (LDHs, Supplementary Fig. 27). This alleviates dendrite formation during Zn plating, as validated by the homogeneous, smooth and dense Zn deposition morphology (Fig. 4d and Supplementary Figs. 28a–c, 29). In 0SL, the high surface area LDHs induce inhomogeneity and aggravate dendrite formation, creating spongy Zn deposits (Fig. 4e and Supplementary Fig. 28d–f).

The Zn SEI chemistry was explored by depth-profiling high-resolution X-ray photoelectron spectroscopy (XPS). In the F *1s* region of Zn deposited in 0SL (Fig. 4f), the CF_3_ species at 688.6 eV originates from OTf^-^, along with the feature in the C *1s* region at 292.8 eV. The OTf^-^ is incorporated in the LDH (Supplementary Fig. 27). Reduction of the OTf^-^ (169.2 eV) anion is signaled by a peak at 684.7 eV ascribed to ZnF_2_^17^; along with sulfites (167.3 eV) and ZnS (162.1 eV) observed in the S *2p* spectra. In the O *1s* region, the two peaks correspond to ZnCO_3_ (532 eV) and ZnO/Zn(OH)_2_ (530.5 eV), as confirmed by their characteristic lattice d-spacings measured by high-resolution transmission electron microscopy (TEM; Fig. 4g). In summary, Zn deposited in 0SL yields a complex interphase that can be characterized as a mixture of ZnCO_3_-ZnO/Zn(OH)_2_ - ZnF_2_ - ZnS - sulfite - LDH. While a ZnF_2_-rich interface has been reported as a protective layer to realize a highly reversible Zn anode^15–18^, nonetheless, the high-porosity LDH reduces the ability to exclude water from the Zn surface.

The XPS spectra of the Zn deposited in 70SL are quite different (Fig. 4h). In particular, no CF_3_ species appear in the C *1s* region and no significant features were observed in the F *1s* region, indicating OTf^-^ reduction does not occur at the interface. Thus, the sulfite and ZnS peaks in the S *2p* region must originate from the reduction of sulfolane rather than OTf^-^. More evidence of inorganic ZnS, ZnCO_3_, and ZnO was obtained from TEM (Fig. 4i). These findings show that an *LDH/ZnF*_*2*_*-free*, ZnCO_3_-ZnO/Zn(OH)_2_-ZnS-sulfite SEI forms at the Zn surface. This SEI not only prevents water penetration but also suppresses the adsorption and reduction of OTf^-^ on the Zn surface, resulting in depressed kinetics of HER and deposition of LDHs, thus enhancing the reversibility and stability of Zn plating/stripping.

### Long-term full-cell performance under practical conditions

**Fig. 5:**
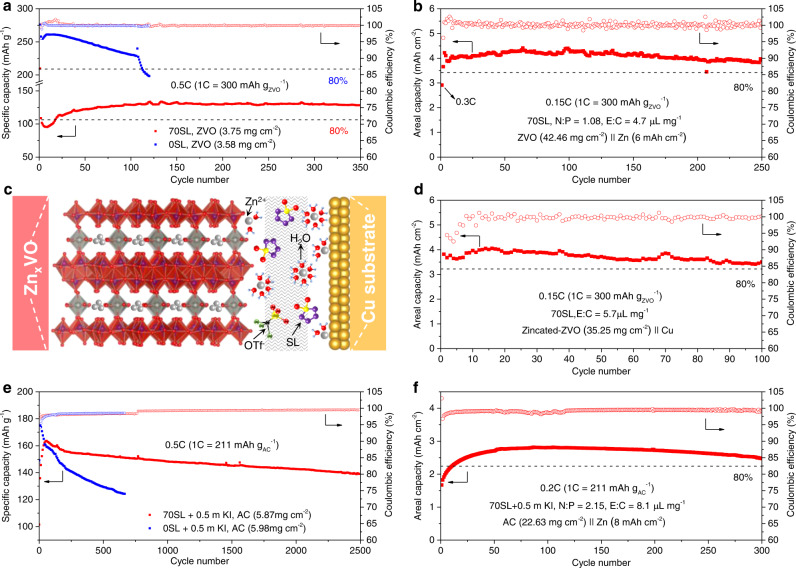
Electrochemical performance of practical full cells. **a** Discharge capacity of ZVO as a function of cycle number in different electrolytes at 0.5 C. **b** Long-term cycling performance of high-loading ZVO in 70SL at 0.15 C and a controlled N/P ratio of 1.08. **c**, **d** Schematic of an anode-free Zn_0.25+x_VO||Cu cell with 70SL (**c**) and corresponding cycling performance at 0.15 C (**d**). **e** Discharge capacity of activated carbon (AC) electrodes as a function of cycle numbers in different electrolytes with the addition of 0.5 m KI at 0.5 C (unexpected power shutdown at 770^th^ cycle). **f** Long-term cycling performance of high-loading AC in 70SL + 0.5 m KI at 0.2 C at a controlled N/P ratio of 2.15.

The dual-stabilized electrodes prompted us to further investigate full-cell performance. In contrast to using high current rates up to 10 to 25 C enabled by a high degree of proton co-intercalation^35–37^, intermediate rates (<1 C) and much higher areal capacities up to 4 mAh cm^−2^ were applied in this work (Fig. 5a–d). These metrics are recognized as one of the major challenges for practical aqueous ZMBs^5^. At 0.5 C and a ZVO cathode loading of ~ 1.1 mAh cm^−2^ (based on 300 mAh g_zvo_^−1^, Fig. 5a and Supplementary Fig. 30) in 0SL, ZVO fades quickly with a decay rate of 0.12% per cycle before short-circuiting at the 106th cycle. This contrasts with its outstanding stability at a 10 C rate with a decay of 0.003% per cycle resulting in ~91% capacity retention after 3000 cycles (Supplementary Fig. 31), similar to previous reports^37,46,53^. This is because, at high rates, the shorter time that the cathode spends at extreme potentials suppresses deterioration owing to vanadium dissolution, proton intercalation, and corresponding LDH formation. In 70SL, these side reactions are effectively addressed, resulting in very good cycling stability of ZVO even at a 0.5 C rate with virtually no capacity decay after 350 cycles. To further address the practical requirements of ZMBs, we fabricated free-standing ZVO electrodes with a high loading of ~42 mg cm^−2^ and a thickness of ~580 μm (Supplementary Fig. 32). In 70SL, full cells based on such high-loading ZVO cathodes provide a practical-level areal capacity of 4.4 mAh cm^−2^ at 0.15 C (Fig. 5b and Supplementary Fig. 33). The cell maintains ~90% capacity retention over 250 cycles at a low negative/positive ratio (N/P) of 1.08 (based on 130 mAh g_zvo_^−1^ achieved at 0.5 C in Fig. 5a) and a low electrolyte volume/active material mass ratio (E/C) of 4.7 μL mg^−1^. Importantly, an average CE of ~100% is obtained. Under even more challenging conditions, an anode-free Zn_0.25+x_VO||Cu cell configuration was constructed, with the only source of Zn being that stored in pre-zincated ZVO (Fig. 5c, d). The cell exhibits an areal capacity of ~4 mAh cm^−2^ and maintains 85% of its capacity after 100 cycles at 0.15 C.

We further verified the adaptability of this electrolyte design strategy with conversion-type Zn||Iodine full cells, which obviate the sluggish Zn^2+^ diffusion in ZVO. In these studies, 0.5 m KI dissolved in 0SL or 70SL was the active material, and high-surface-area activated carbon (AC) cathode acted as the iodine host (Fig. 5e and Supplementary Fig. 34). The reversible capacity in 70SL is 164 mAh g_AC_^−1^ at 0.5 C (1 C = 212 mA g_AC_^−1^), only slightly lower than in 0SL (175 mAh g_AC_^−1^), and indicating better kinetics compared to intercalative ZVO. The Zn||iodine cell with 70SL also shows excellent stability at a high rate of 10 C (Supplementary Fig. 35). Moreover, the reduced water activity in 70SL suppresses the dissolution of I_2_ and formation of the intermediate I_3_^-^ at charged states^54^, leading to much-improved cycling stability with a capacity retention of 86% over 2500 cycles, as opposed to only 70% after 670 cycles in 0SL. A practical-level full cell with a high-loading AC cathode (Supplementary Fig. 36) at an N/P ratio of 2.15 (based on 164 mAh g_AC_^−1^ achieved at 0.5 C in Fig. 5e) and E/C ratio of 8.1 μL mg^−1^ (Fig. 5f and Supplementary Fig. 37), provided an areal capacity of 2.8 mAh cm^−2^ with 88% capacity retention after 300 cycles at 0.2 C.

## Discussion

The competition between Zn^2+^ vs. proton intercalation in a conventional ZVO cathode is shifted to 95% Zn^2+^-selective with a high-sulfolane/water hybrid eutectic electrolyte that alleviates deleterious proton intercalation and cathode dissolution. Dominant Zn^2+^ intercalation—disclosed by multiple physical/electrochemical characterization techniques—stabilizes the ZVO at low and moderate current densities with a high-areal capacity of over 4 mAh cm^−2^. Simultaneously, a fully hydrated Zn^2+^ solvation structure—revealed by a combination of experimental and theoretical characterization—enables a facile interfacial desolvation process at the solid/electrolyte interface. We realize highly reversible Zn plating/stripping with a 20-fold extended lifetime assisted by a sulfolane-derived SEI, and demonstrate excellent low-rate cycling stability of practical-level *anode-free* cells. We do acknowledge that while dominant Zn^2+^ intercalation chemistry stabilizes the low or intermediate-rate performance necessary for long-duration energy storage, it inevitably diminishes ultra-high-rate performance needed for high-power load leveling. Solid-state Zn^2+^-diffusion is slower than H^+^ diffusion in hydrated oxides. This highlights that developing a new generation of cathode materials that are amenable to Zn desolvation/solvation (without proton co-intercalation) is likely necessary to realize high-rate function while not compromising the low-rate stability. Our work represents a practical strategy to stabilize both electrodes by tuning the hybrid electrolyte network and also provides an understanding of Zn^2+^-(de)intercalation in conventional oxide cathodes which could expedite the commercialization of practical ZMBs.

## Methods

### Preparation of hybrid eutectic electrolytes

Hybrid eutectic co-solvents were prepared by mixing 0.3, 0.5, 0.8, and 1.0 g deionized water with 0.7, 0.5, 0.2, and 0 g sulfolane (99%, Sigma-Aldrich) at 25 °C. Subsequently, 2 mmol Zn(OTf)_2_ (98%, Sigma-Aldrich) was added into the above mixtures to obtain the 70SL, 50SL, 20SL, and 0SL electrolytes, respectively. For Zn-iodine full-cell measurements, 0.5 m KI was added into 70SL and 0SL as the active material.

### Preparation of Zn_0.25_V_2_O_5_·nH_2_O (ZVO) and activated carbon (AC) cathodes

The ZVO nanobelts were synthesized based on a previously reported microwave hydrothermal method^46^. Typically, 2 mmol V_2_O_5_ (99.6%; Sigma-Aldrich) and 1.3 mmol Zn(CH_3_COO)_2_·2H_2_O (99%; Sigma-Aldrich) were mixed in 50 ml water/acetone (volume ratio: 15:1) solution. The mixture was transferred to a sealed Teflon vessel and heated at 18 °C min^−1^ in an Anton Parr microwave (Synthos 300), and then maintained at 180 °C for 90 min. The resulting Zn_0.25_V_2_O_5_·nH_2_O nanobelts were washed with deionized water and isopropanol, and then dried in a 60 °C oven for 24 h. High-surface area Black Pearls 2000 carbon (BP 2000) was directly used as the iodine host.

The low-loading cathodes were prepared by mixing 70 wt% active material, 20 wt% conductive carbon (Super P), and 10 wt% polyvinylidene difluoride (PVDF) in *N*-methyl-2-pyrrolidone (NMP; anhydrous, 99.5%; Sigma-Aldrich). The mixed homogeneous slurry was cast on carbon paper (thickness: 170 μm) by doctor blading. The electrodes were punched into 10 mm disks after being dried at 60 °C overnight. The mass loading of active material was approximately 3.6 ± 0.3 mg cm^−2^, although each cathode laminate was weighed independently. To prepare free-standing thick electrodes, 80 wt% active materials were mixed with 10 wt% conductive carbon (Ketjenblack) and 10 wt% polytetrafluoroethylene (PTFE, 60 wt% in H_2_O, Sigma-Aldrich), using isopropanol (99.5%, Sigma-Aldrich) as the dispersing agent. The slurry thus obtained was quickly pressed to form free-standing electrodes. After drying at 60 °C overnight, the electrodes were cut into small pieces and pressed onto 10 mm titanium mesh.

### Electrochemical measurements

PFA-based Swagelok-type cells were used in this study. The Zn||Cu asymmetric cells were assembled using Cu foil (thickness: 9 μm; diameter: 11 mm) as the positive current collector, Zn metal foil (99.9%; Sigma-Aldrich; thickness: 250 μm; diameter: 10 mm) as the negative, and commercial Whatman glass fiber as the separator (diameter: 12.7 mm). In Zn||Zn symmetric cells, Zn foils were used as both positive and negative electrodes. The full cells with low-loading ZVO or activated carbon (AC) cathodes were assembled with 100 μL electrolyte, using Zn metal foil as the anode. Cu foil with pre-deposited Zn was utilized as the anode in high-loading full cells, where the amount of electrolyte was increased to 200 μL to ensure good electrode infusion. The electrochemical stability window of the hybrid eutectic electrolyte was investigated by linear sweep voltammetry (LSV) at a sweep rate of 1 mV s^−1^, based on three-electrode cells with Zn as the reference electrode, and Ti as both working and counter electrodes. Chronoamperometry (CA) measurements were conducted at a constant polarization of 5 mV on Zn||Zn symmetric cells. All cells were cycled using a VMP3 potentiostat/galvanostat station (Bio-Logic) at 25 °C.

### Impedance analysis

The evolution of charge transfer resistance of two-electrode Zn||Zn symmetric cells was measured by electrochemical impedance spectroscopy (EIS) with a voltage amplitude of 5 mV in the frequency range of 1 MHz to 100 mHz. The cell was placed at rest for 10 min to reach equilibrium before each EIS measurement. For the activation energy measurements, three-electrode cells were assembled with Zn as both working, counter, and reference electrodes. Impedance was measured from 1 MHz to 1 Hz with 10 mV amplitude, at temperatures ranging from 40 to 70 °C. The activation energy (*Ea*) for interfacial charge transfer is extrapolated based on the Arrhenius Eq. (1):

1 $$\frac{1}{{R}_{{{{{{{\mathrm{CT}}}}}}}}}=A\,{e}^{-\frac{{E}_{a}}{{RT}}}$$

where *R*_CT_ is the charge transfer resistance, *R* is the ideal gas constant, *T* is the absolute temperature, and *A* is the frequency factor.

### Galvanostatic intermittent titration technique (GITT) studies

GITT of ZVO in different electrolytes were performed using a current pulse of 20 min at an equivalent 0.1 C rate followed by a 3-hour rest. The average diffusion coefficients (*D*_*eff*_) in ZVO were calculated from the GITT data by the following Eq. (2) first reported by Weppner and Huggins^55^:

2 $$D=\frac{4}{\pi \tau }\times {\left(\frac{{n}_{M}{V}_{M}}{S}\right)}^{2}\times {\left(\frac{{\Delta E}_{s}}{\Delta {E}_{t}}\right)}^{2}$$

where τ is the current pulse duration (20 min) of a single step of the GITT experiment; *ΔE*_*S*_ and *ΔE*_*t*_ are the change in the steady state voltage and overall cell voltage in one current pulse, respectively; *n*_*M*_ and *V*_*M*_ are the moles and molar volume of ZVO, respectively; and the geometric area (*S*) of the electrode is used for simplicity and comparison with previous data.

### Material characterization

Powder X-ray diffraction (XRD) patterns were obtained on an X-ray diffractometer (PANalytical Empyrean) with Cu-Kα radiation and a PIXcel bidimensional detector. *Operando* XRD measurements were performed in a home-built cell, using glassy carbon as the current collector and X-ray window. Free-standing electrodes were used in these *operando* diffraction experiments, where low-angle peaks (<10°, 2θ) were blocked by the cell case. Fourier transform infrared spectroscopy (FTIR) was carried out on a Bruker Tensor 27 system equipped with attenuated total reflection (ATR). ^1^H-NMR experiments were carried on a Bruker Advance 300 MHZ instrument at 25 °C. The ionic conductivity and viscosity of the series of electrolytes was measured using an Orion Star A325 pH/Conductivity Meter (Thermo) and an automated viscometer (RheoSense Inc.) at 22 °C, respectively. To collect X-ray absorption near-edge structure (XANES) and the Fourier transformation of extended X-ray absorption fine structure (EXAFS) spectra, samples were prepared by wetting multiple layers of glass fiber separator that were packaged between two glass coverslips with a fluorosilicone gasket. Dilute samples were measured in 3 mm NMR tubes. In both cases, X-ray absorption was measured in transmission mode using a zinc metal foil reference (9659 eV). A Zeiss Ultra field emission SEM instrument was used to collect scanning electron microscopy (SEM) images and energy-dispersive X-ray spectroscopy (EDS) data. The in situ optical microscopy study was carried out using a homemade transparent electrochemical cell and an optical microscope outfitted with a digital camera. XPS experiments were conducted on a Thermo Scientific K-Alpha XPS instrument. CasaXPS software was used to conduct XPS data analysis, where the C 1*s* peak of 284.8 eV was used to calibrate the binding energies. Spectral fitting was based on Gaussian–Lorentzian functions and a Shirley-type background. The Ar^+^-ion sputtering etching rate was estimated to be ~1.0 nm min^−1^. High-resolution transmission electron microscopy (TEM) images of deposited Zn were collected on a Hitachi HF-3300. All electrodes disassembled from the cells were washed with water five times and by ethanol twice and dried at 25 °C before each characterization. Differential scanning calorimetry (DSC) was performed on Netzsch STA 449 F3 Jupiter from −100 to 25 °C at a ramp rate of 10 °C min^−1^. Gas chromatography (GC) data were obtained on a multiple gas analyzer SRI MG-5 using Ar as the gas carrier.

### Scanning transmission electron microscopy (STEM)

The high-resolution imaging and spectroscopy experiments were conducted at the University of Illinois Chicago using a JEOL JEM-ARM200CF microscope operating at 200 kV. The microscope is equipped with a cold-field emission electron gun and a CEOS aberration corrector with a probe size of 0.78 Å. The pristine and discharged samples were dispersed in acetonitrile and subsequently drop-cast onto a TEM grid. EDS data was acquired using the Oxford X-Max 100TLE windowless silicon drift detector. EELS experiments were carried out using a dual-range Gatan Continuum spectrometer with a dispersion of 0.3 eV per channel and an entrance aperture of 5 mm. The convergence and collection semi-angles for the spectrometer were 17.8 mrad and 53.4 mrad, respectively. The zero-loss peak (ZLP) and the core-loss edges (V L, O K, and Zn L edges) were acquired simultaneously with an acquisition time of 0.0001 and 0.25 s per pixel, respectively. Fourier log deconvolution was performed to remove the multiple scattering components from the core-loss edges. To improve the signal-to-noise ratio of the core-loss spectra, we performed a principal component analysis. A power law was used to model the background signal before V L and Zn L edges. The atomic-resolution HAADF images were obtained using the probe convergence angle of 25 mrad where the inner and outer diameters of the annular detector were set to 68 mrad and 174.5 mrad. To minimize electron beam damage, the atomic-resolution HAADF imaging was performed sequentially, where multiple frames (10–20 frames) were aligned and integrated to obtain atomically resolved HAADF images with a high signal-to-noise ratio. The electron dose was kept below a threshold of 400 e/Å^2^ for the pristine and discharged ZVO samples during STEM experiments.

### Molecular dynamics simulations

Classical MD simulations of the bulk electrolyte were used to generate solvated clusters for subsequent DFT and XAS calculations, and to examine changes in solvation structure and free water content with SL loading. Initial configurations were generated by packing the appropriate number of molecules into a simulation box with packmol^56^. Following energy minimization, we confirmed that the equilibrated box size was at least 50 Å per side, which was more than twice the Lennard-Jones cutoff distance. Subsequent simulation steps comprised 2 ns in the NPT ensemble at 1 bar and 600 K, followed by 2 ns cooling from 600 to 298 K and 1 ns at 298 K. After this initial equilibration, dynamics were collected in the NVT ensemble for 5 ns. All simulations were carried out using LAMMPS^57^.

We used the TIP4P-FB water model^58^ with corresponding Zn^2+^ ion parameters^59^ designed to reproduce the ion-oxygen coordination distance with high accuracy (the IOD set in the original reference). For OTf^-^ and SL molecules we used generalized amber force field (GAFF)^60^ parameters with AM1-BCC partial charges. Next, charges on Zn^2+^ and the OTf^-^ ions were scaled by a factor of 0.8, which is an established method of correcting errors in electronic polarization effects that arise in non-polarizable water models^44,45,61^. Because the coordinating oxygen species on OTf^-^ and SL used the same (GAFF) force field parameters, we also applied the 0.8 charge scaling factor to the partial charges on the SL molecule to preserve the relative strength of OTf-O and SL-O interactions with Zn^2+^. Scaling partial charges on solvent molecules has been used to calibrate simulated transport properties in other mixed solvent electrolytes^62,63^. In our case, we found it necessary to scale the SL charges by the same amount as the ion charges in order to reproduce both the predominance of fully hydrated Zn^2+^ in (1) 0SL electrolytes, which is well-established by experiments^64^ and (2) higher SL loadings, which was indicated by first-principles DFT free energy calculations and experimental and computed XAS spectra. In the absence of scaling the SL charges, our classical MD simulations predicted a significant amount of SL coordination of the Zn^2+^ ions (see Supplementary Fig. 38).

Analysis of solvation shells and water populations was performed using the solvation-analysis^65^ plugin for MDAnalysis^66,67^ according to methods we reported in previous work^12^. For each species coordinating Zn^2+^ (H_2_O-O, OTf-O, and SL-O) we determined coordination cutoff radii from the radial distribution function (Supplementary Fig. 39), then used analyzed the relative frequency of different solvation configurations. We used a similar methodology to determine the fraction of free water (i.e., water not coordinated to Zn^2+^, OTf^-^, or SL).

### Density functional theory simulations

Solvated clusters comprising a single Zn^2+^ and all molecules in the first hydration shell were extracted from the MD simulations and used as the starting configurations for DFT calculations of free energy. The same calculation protocol was performed for clusters containing between 1 and 12 H_2_O molecules, a single OTf^-^ molecule, and a single sulfolane molecule. We first optimized the structures using the ωB97X-D^68^ functional with def2-SVPD^69^ basis and a PCM implicit dielectric of 78.4. After initial optimization, we utilized the frequency flattening optimization workflow implemented in atomate^70^ to obtain vibrational frequencies at the same level of theory (ωB97X-D/def2-SVPD/PCM). This workflow first performs a frequency calculation, then checks whether there are any imaginary frequency modes present. If imaginary modes are found, the geometry is perturbed slightly and re-relaxed. The process is repeated until there are no imaginary frequencies remaining, indicating that a true minimum on the potential energy surface has been found. Following the successful frequency flattening procedure, we then performed a single-point energy calculation on the final geometry using the ωB97X-V^71^ functional with def2-TZVPD^72^ basis set and the SMD^73^ implicit solvent model, with water as the solvent. All calculations were performed in Q-Chem^74^ version 5.4.

The free energy of each molecule was obtained from (1) the electronic energy of the single-point energy calculation and (2) the enthalpy and entropy obtained from the vibrational frequencies. We found that the free energy per H_2_O molecule converged with increasing water cluster size, presumably due to more precise handling of inter-molecule dispersion effects, and hence in our calculations, the free energy per H_2_O molecule was taken from a cluster with *n* = 12 water molecules rather than from a single water molecule (see Supplementary Fig. 40).

Having obtained the free energy of the solvated Zn^2+^ clusters and the respective molecules, we computed the free energy of the reaction to form fully hydrated Zn^2+^ (i.e., Zn^2+^·*6*H_2_O) from either Zn^2+^·*4*H_2_O·*2*OTf^-^ or Zn^2+^·*4*H_2_O·*1*OTf^-^·*1*SL, according to the following reaction (3) and (4):

3 $${{{{{\mathrm{Z}}}}}}{{{{{{\mathrm{n}}}}}}}^{2+} \cdot 4{{{{{{\mathrm{H}}}}}}}_{2}{{{{{\mathrm{O}}}}}}\cdot 2{{{{{{\mathrm{OT}}}}}}}{f}^{-}+{{{{{\bf{2}}}}}}{{{{{{\mathrm{H}}}}}}}_{2}{{{{{\mathrm{O}}}}}}\to {{{{{\mathrm{Z}}}}}}{{{{{{\mathrm{n}}}}}}}^{2+}\cdot 6{{{{{{\mathrm{H}}}}}}}_{2}{{{{{\mathrm{O}}}}}}+{{{{{\bf{2}}}}}}{{{{{{\mathrm{OT}}}}}}}{f}^{-}$$

4 $${{{{{\mathrm{Z}}}}}}{{{{{{\mathrm{n}}}}}}}^{2+} \cdot 4{{{{{{\mathrm{H}}}}}}}_{2}{{{{{\mathrm{O}}}}}}{\cdot }1{{{{{{\mathrm{OT}}}}}}}{f}^{-}{\cdot }1{{{{{{\mathrm{SL}}}}}}}+{{{{{\bf{2}}}}}}{{{{{{\mathrm{H}}}}}}}_{2}{{{{{\mathrm{O}}}}}}\to {{{{{\mathrm{Z}}}}}}{{{{{{\mathrm{n}}}}}}}^{2+}\cdot 6{{{{{{\mathrm{H}}}}}}}_{2}{{{{{\mathrm{O}}}}}}+{{{{{\bf{1}}}}}}{{{{{{\mathrm{OT}}}}}}}{f}^{-}+{{{{{\bf{1}}}}}}{{{{{{\mathrm{SL}}}}}}}$$

### Simulated X-ray absorption (XANES) spectra

K-edge X-ray absorption near-edge structure (XANES) spectra arising from specific solvation configurations were simulated from first principles using the FEFF10 software^75^ via the XANES workflow implemented in atomate^70^. Zn^2+^ atoms having a particular solvated configuration were identified from the MD trajectories as described above, and these were treated as absorbing atoms. To avoid periodic boundary effects, only Zn^2+^ atoms that were at least 7.5 Å from the edge of the simulation cell were considered. Then, all neighboring molecules within 7.5 Å of this central Zn^2+^ were extracted from the MD trajectory and used to compute the spectrum. The 7.5 Å radius was chosen to correspond to the RFMS flag of FEFF, which sets how many atoms are considered in the calculation. Calculations were performed in real space using one unique potential per element, an SCF radius of 5 Å (large enough to encompass the second coordination shell, as determined by radial distribution functions from the MD trajectories), and the RPA method for screening the core-hole potential. Other calculation parameters were those established and benchmarked for convergence over a set of crystalline materials by refs. ^76,77^ during the development of the atomate workflow. The spectra we report represent the average of 5 independent replicates of each solvated configuration. The resulting spectra were shifted horizontally by 0–19 eV so that the primary peak for all clusters coincides with that of fully hydrated Zn^2+^ at 9685 eV.

## Supplementary information

Supplementary Information

Peer review file

Description to Additional Supplementary Information

Supplementary Movie 1

Supplementary Movie 2

## Data Availability

All relevant data that support the findings of this study are presented in the article and Supplementary Information. Source data are available from the corresponding authors upon request.

## References

[1] Liu Z (2020). Voltage issue of aqueous rechargeable metal-ion batteries. Chem. Soc. Rev..

[2] Blanc EL, Kundu D, Nazar LF (2020). Scientific challenges for the implementation of Zn-ion batteries. Joule.

[3] Park MJ, Asl HY, Manthiram A (2020). Multivalent-ion versus proton insertion into battery electrodes. ACS Energy Lett..

[4] Oberholzer P, Tervoort E, Bouzid A, Pasquarello A, Kundu D (2019). Oxide versus nonoxide cathode materials for aqueous Zn batteries: an insight into the charge storage mechanism and consequences thereof. ACS Appl. Mater. Interfaces.

[5] Li C, Jin S, Archer LA, Nazar LF (2022). Towards practical aqueous zinc-ion batteries for electrochemical energy storage. Joule.

[6] Ma L (2020). Realizing high zinc reversibility in rechargeable batteries. Nat. Energy.

[7] Liu S (2022). Suppressing vanadium dissolution by modulating aqueous electrolyte structure for ultralong lifespan zinc ion batteries at low current density. Energy Storage Mater..

[8] Liu X (2019). *Operando* pH measurements decipher H^+^/Zn^2+^ intercalation chemsitry in high-performance aqueous Zn/β-V_2_O_5_ batteries. ACS Energy Lett..

[9] Yuan Y (2022). Understanding intercalation chemistry for sustainable aqueous zinc-manganese dioxide batteries. Nat. Sustain..

[10] Pan H (2016). Reversible aqueous zinc/manganese oxide energy storage from conversion reactions. Nat. Energy.

[11] Albertus P, Manser JS, Litzelman S (2020). Long-duration electricity storage applications, economics, and technologies. Joule.

[12] Li C (2022). Tuning the solvation structure in aqueous zinc batteries to maximize Zn-ion intercalation and optimize dendrite-free zinc plating. ACS Energy Lett..

[13] Wang F (2021). Quantifying and suppressing proton intercalation to enable high-voltage Zn-ion batteries. Adv. Energy Mater..

[14] Zheng J (2019). Reversible epitaxial electrodeposition of metals in battery anodes. Science.

[15] Liang G (2022). Gradient fluorinated alloy to enable highly reversible Zn-metal anode chemistry. Energy Environ. Sci..

[16] Ma L (2021). Toward practical high-areal-capacity aqueous zinc-metal batteries: quantifying hydrogen evolution and a solid-ion conductor for stable zinc anodes. Adv. Mater..

[17] Cao L (2021). Fluorinated interphase enables reversible aqueous zinc battery chemistries. Nat. Nanotechnol..

[18] Han D (2021). A non-flammable hydrous organic electrolyte for sustainable zinc batteries. Nat. Sustain..

[19] Cao L (2020). Solvation structure design for aqueous Zn metal batteries. J. Am. Chem. Soc..

[20] Li C (2022). Highly reversible Zn anode with a practical areal capacity enabled by a sustainable electrolyte and superacid interfacial chemistry. Joule.

[21] Wang Z (2019). A metal-organic framework host for highly reversible dendrite-free zinc metal anodes. Joule.

[22] Parker JF (2017). Rechargeable nickel-3D zinc batteries: an energy-dense, safer alternative to lithium-ion. Science.

[23] Chamoun M (2015). Hyper-dendritic nanoporous zinc foam anodes. NPG Asia Mater..

[24] Wang F (2018). Highly reversible zinc metal anode for aqueous batteries. Nat. Mater..

[25] Zhang C (2018). A ZnCl_2_ water-in salt electrolyte for a reversible Zn metal anode. Chem. Commun..

[26] Xie J, Liang Z, Lu Y (2020). Molecular crowding electrolytes for high-voltage aqueous batteries. Nat. Mater..

[27] Wang Y (2022). Enabling high-energy-density aqueous batteries with hydrogen bond-anchored electrolytes. Matter.

[28] Liu J (2022). Water/sulfolane hybrid electrolyte achieves ultralow-temperature operation for high-voltage aqueous lithium-ion batteries. Adv. Funct. Mater..

[29] Cheng X (2022). 2.5V high-performance aqueous and semi-solid-state symmetric supercapacitors enabled by 3m sulfolane-saturated aqueous electrolytes. Energy Technol..

[30] Yang W (2020). Hydrated eutectic electrolytes with ligand-oriented solvation shells for long-cycling zinc-organic batteries. Joule.

[31] Hao J (2021). Boosting Zn electrode reversibility in aqueous electrolytes using low-cost antisolvents. Angew. Chem. Int. Ed..

[32] Chang N (2020). An aqueous hybrid electrolyte for low-temperature zinc-based energy storage devices. Energy Environ. Sci..

[33] Ma G (2022). Non-flammable, dilute, and hydrous organic electrolytes for reversible Zn batteries. Chem. Sci..

[34] Lin X (2021). Hydrated deep eutectic electrolytes for high-performance Zn-ion batteries capable of low-temperature operation. Adv. Funct. Mater..

[35] Zhao X (2022). Advanced buffering acidic aqueous electrolytes for ultra-long life aqueous zinc-ion batteries. Small.

[36] Wang M (2023). High-capacity zinc anode with 96% utilization rate enabled by solvation structure design. Angew. Chem. Int. Ed..

[37] Li, M. et al. Comprehensive H_2_O molecules regulation via deep eutectic solvents for ultra-stable zinc metal anode. *Angew. Chem. Int. Ed*. **62**, e202215552 (2022). 10.1002/anie.202215552 36536537

[38] Ma L, Vatamanu J, Hahn NT, Xu K (2022). Highly reversible Zn metal anode enabled by sustainable hydroxyl chemistry. Proc. Natl Acad. Sci. USA.

[39] Kundu D (2018). Aqueous vs. nonaqueous Zn-ion batteries: consequences of the desolvation penalty at the interface. Energy Environ. Sci..

[40] Zhang N (2016). Cation-deficient spinel ZnMn_2_O_4_ cathode in Zn(CF_3_SO_3_)_2_ electrolyte for rechargeable aqueous Zn-ion battery. J. Am. Chem. Soc..

[41] Zhang N (2017). Rechargeable aqueous zinc-manganese dioxide batteries with high energy and power densities. Nat. Commun..

[42] Yuan D (2021). Anion texturing towards dendrite-free Zn anode for aqueous rechargeable batteries. Angew. Chem. Int. Ed..

[43] Zhang Q (2021). Designing anion-type water-free Zn^2+^ solvation structure for robust Zn metal anode. Angew. Chem. Int. Ed..

[44] Kirby BJ, Jungwirth P (2019). Charge scaling manifesto: a way of reconciling the inherently macroscopic and microscopic natures of molecular simulations. J. Phys. Chem. Lett..

[45] Leontyev I, Stuchebrukhov A (2011). Accounting for electronic polarization in non-polarizable force fields. Phys. Chem. Chem. Phys..

[46] Kundu D, Adams BD, Duffort V, Vajargah SH, Nazar LF (2016). A high-capacity and long-life aqueous rechargeable zinc battery using a metal oxide intercalation cathode. Nat. Energy.

[47] Yoo HD (2019). Intercalation of magnesium into a layered vanadium oxide with high capacity. ACS Energy Lett..

[48] Mukherjee A (2017). Direct characterization of the Li intercalation mechanism into α-V_2_O_5_ nanowires using in-situ transmission electron microscopy. Appl. Phys. Lett..

[49] Varela M (2009). Atomic‐resolution imaging of oxidation states in manganites. Phys. Rev. B.

[50] Mishra R (2016). Towards spin‐polarized two‐dimensional electron gas at a surface of an antiferromagnetic insulating oxide. Phys. Rev. B.

[51] Li Q, Chen A, Wang D, Pei Z, Zhi C (2022). “Soft shorts” hidden in zinc metal anode research. Joule.

[52] Park J, Park S, Joung D, Kim C (2022). Sustainable biopolymeric hydrogel interphase for dendrite-free aqueous zinc-ion batteries. Chem. Eng. J..

[53] Wang W (2022). Ultralow-water-activity electrolyte endows vanadium-based zinc-ion batteries with durable lifespan exceeding 30 000 cycles. Energy Storage Mater..

[54] Yang Y, Liang S, Lu B, Zhou J (2022). Eutectic electrolyte based on N-methylacetamide for highly reversible zinc-iodine battery. Energy Environ. Sci..

[55] Weppner W, Huggins RA (1977). Determination of the kinetic parameters of mixed conducting electrodes and application to the system Li_3_Sb. J. Electrochem. Soc..

[56] Martínez L, Andrade R, Birgin EG, Martínez JM (2009). PACKMOL: a package for building initial configurations for molecular dynamics simulations. J. Comput. Chem..

[57] Thompson AP (2021). LAMMPS - a flexible simulation tool for particle-based materials modeling at the atomic, meso, and continuum scales. Comput. Phys. Commun..

[58] Wang L-P, Martinez TJ, Pande VS (2014). Building force fields: an automatic, systematic, and reproducible approach. J. Phys. Chem. Lett..

[59] Li Z, Song LF, Li P, Merz KM (2020). Systematic parametrization of divalent metal ions for the OPC3, OPC, TIP3P-FB, and TIP4P-FB water models. J. Chem. Theory Comput..

[60] Wang J, Wolf RM, Caldwell JW, Kollman PA, Case DA (2004). Development and testing of a general amber force field. J. Comput. Chem..

[61] Ringsby AJ (2021). Transport phenomena in low temperature lithium-ion battery electrolytes. J. Electrochem. Soc..

[62] Chaudhari MI (2016). Scaling atomic partial charges of carbonate solvents for lithium ion solvation and diffusion. J. Chem. Theory Comput..

[63] Gudla H, Zhang C, Brandell D (2020). Effects of solvent polarity on Li-ion diffusion in polymer electrolytes: an all-atom molecular dynamics study with charge scaling. J. Phys. Chem. B.

[64] Powell DH, Gullidge PMN, Neilson GW, Bellissent-Funel MC (1990). Zn^2+^ hydration and complexation in aqueous electrolyte solutions. Mol. Phys..

[65] Cohen, O. Solvation-analysis. (2021).

[66] Gowers, R. et al. MDAnalysis: a python package for the rapid analysis of molecular dynamics simulations. In *Proc. of the 15th Python in Science Conference* (*SCIPY 2016*) 98–105 (2016).

[67] Michaud-Agrawal N, Denning EJ, Woolf TB, Beckstein O (2011). MD analysis: a toolkit for the analysis of molecular dynamics simulations. J. Comput. Chem..

[68] Chai J-D, Head-Gordon M (2008). Long-range corrected hybrid density functionals with damped atom–atom dispersion corrections. Phys. Chem. Chem. Phys..

[69] Rappoport D, Furche F (2010). Property-optimized Gaussian basis sets for molecular response calculations. J. Chem. Phys..

[70] Mathew K (2017). Atomate: A high-level interface to generate, execute, and analyze computational materials science workflows. Comput. Mater. Sci..

[71] Mardirossian N, Head-Gordon M (2014). ωB97X-V: a 10-parameter, range-separated hybrid, generalized gradient approximation density functional with nonlocal correlation, designed by a survival-of-the-fittest strategy. Phys. Chem. Chem. Phys..

[72] Weigend F, Ahlrichs R (2005). Balanced basis sets of split valence, triple zeta valence and quadruple zeta valence quality for H to Rn: Design and assessment of accuracy. Phys. Chem. Chem. Phys..

[73] Marenich AV, Cramer CJ, Truhlar DG (2009). Universal solvation model based on solute electron density and on a continuum model of the solvent defined by the bulk dielectric constant and atomic surface tensions. J. Phys. Chem. B.

[74] Shao Y (2015). Advances in molecular quantum chemistry contained in the Q-Chem 4 program package. Mol. Phys..

[75] Kas JJ, Vila FD, Pemmaraju CD, Tan TS, Rehr JJ (2021). Advanced calculations of X-ray spectroscopies with FEFF10 and Corvus. J. Synchrotron Radiat..

[76] Zheng C (2018). Automated generation and ensemble-learned matching of X-ray absorption spectra. Npj Comput. Mater..

[77] Mathew K (2018). High-throughput computational X-ray absorption spectroscopy. Sci. Data.

