# Supplementary material for: Enabling selective zinc-ion intercalation by a eutectic electrolyte for practical anodeless zinc batteries
Source: Nat Commun. 2023 May 27;14:3067. doi: 10.1038/s41467-023-38460-2 (PMC10224959; doi:10.1038/s41467-023-38460-2)
Supplement: Supplementary file 1 — Supplementary Information [file 41467_2023_38460_MOESM1_ESM.pdf]

Supplementary Information for:

Enabling Selective Zinc-Ion Intercalation by a Eutectic Electrolyte for Practical Anodeless Zinc Batteries

Chang Li,[a, b] Ryan Kingsbury,[c] Arashdeep Singh Thind,[b, d] Abhinandan Shyamsunder,[a, b] Timothy T. Fister,[b, e] Robert F. Klie, [b, d] Kristin A. Persson,[b, f, g] and Linda F. Nazar* [a, b]

[a] Department of Chemistry and the Waterloo Institute for Nanotechnology, University of Waterloo, Ontario N2L 3G1, Canada

[b] Joint Center for Energy Storage Research, Argonne National Laboratory, Lemont, Illinois 60439, United States

[c] Energy Storage and Distributed Resources Division, Lawrence Berkeley National Laboratory, 1 Cyclotron Road, Berkeley, California 94720, United States

[d] Department of Physics, University of Illinois - Chicago, Chicago, Illinois 60607, United States

[e] Chemical Sciences and Engineering Division, Argonne National Laboratory, Lemont IL 60439, United States

[f] Molecular Foundry, Lawrence Berkeley National Laboratory, 1 Cyclotron Road, Berkeley, California 94720, United States

[g] Department of Materials Science and Engineering, UC Berkeley, Berkeley, California 94720, United States

Corresponding Author:

*Linda F. Nazar: lfnazar@uwaterloo.ca

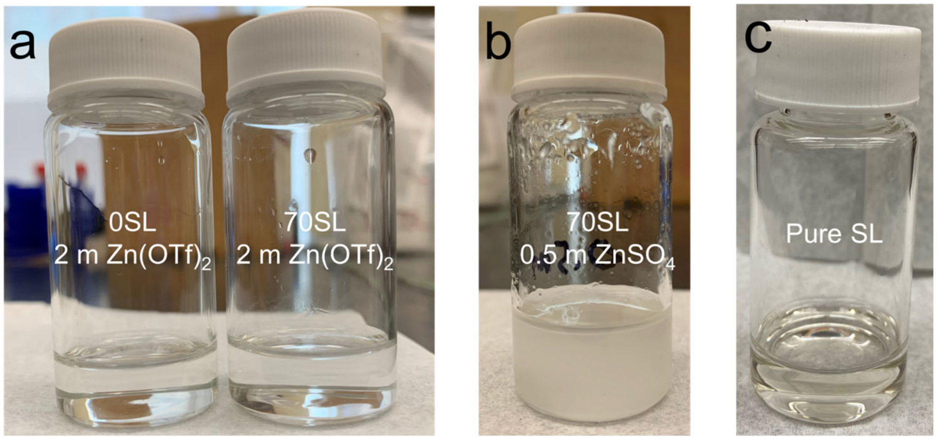

Supplementary Fig. 1 | Pictures of prepared hybrid eutectic electrolyte. **a** 0SL/2 m $Zn(OTf)_2$ and 70SL/2 m $Zn(OTf)_2$ shows similar colorless electrolyte with all salts fully dissolved. **b** 70SL/0.5 m $ZnSO_4$ shows a cloudy mixture, indicating very limited solubility of $ZnSO_4$. **c** Pure SL shows slightly yellowish color due to the unknown industrial impurity, which could have a possible but minor effect on electrolyte properties considering its very low quantity (<1% in pure SL). All electrolytes were stirred at room temperature for at least 4 hours to fully mix solvents and salts.

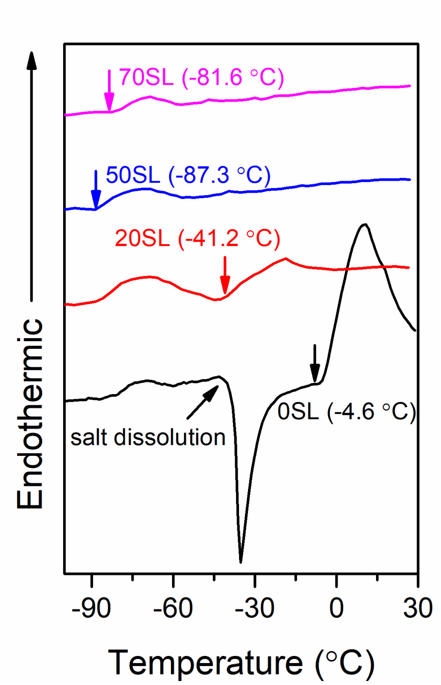

Supplementary Fig. 2 | DSC curves with labeled melting points of various electrolytes. Data were collected from -100 °C to room temperature with a heating rate of 10 °C min^{-1}.

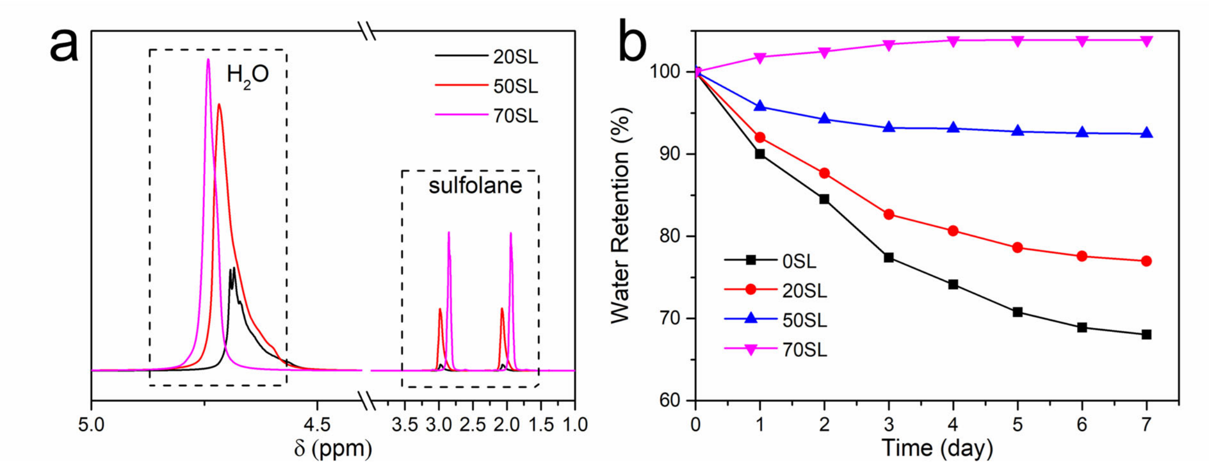

Supplementary Fig. 3 | a ^{1}H-NMR spectra of different electrolytes. **b** water retention of different electrolytes as a function of time on exposure to open atmosphere.

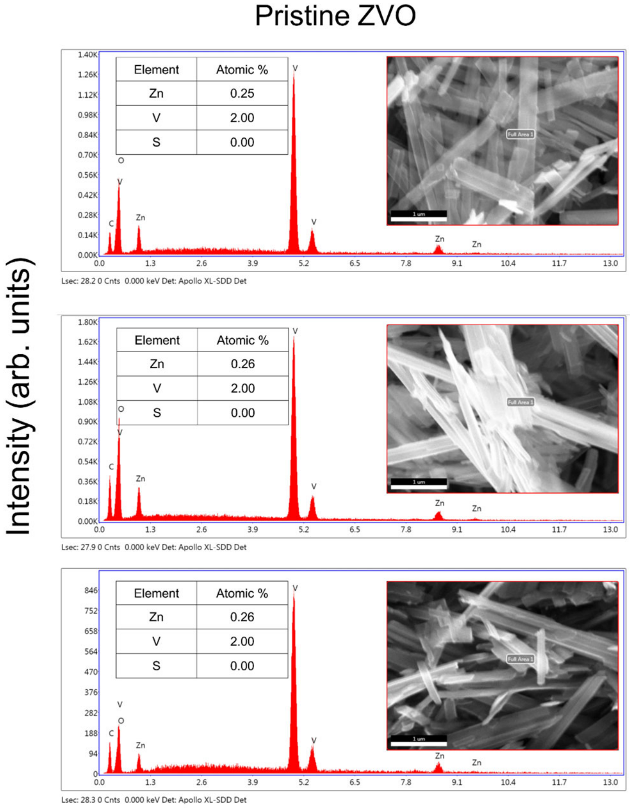

Supplementary Fig. 4 | Three representative samples of EDS data for the pristine ZVO cathode. All atomic percentage are normalized by $Zn_{0.25}V_2O_5{\cdot}nH_2O$ formula with V ratio fixed as 2.

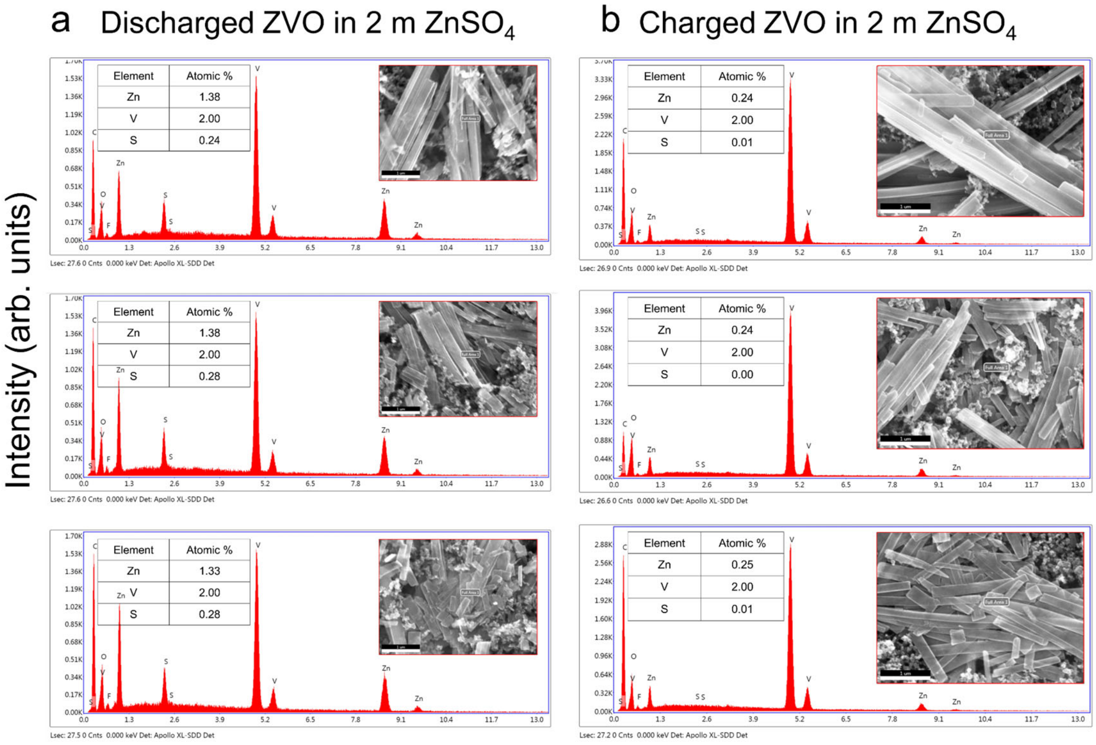

Supplementary Fig. 5 | Three representative samples of EDS data for the **(a)** discharged and **(b)** charged ZVO cathode in 2 m $ZnSO_4$ at 0.5C.

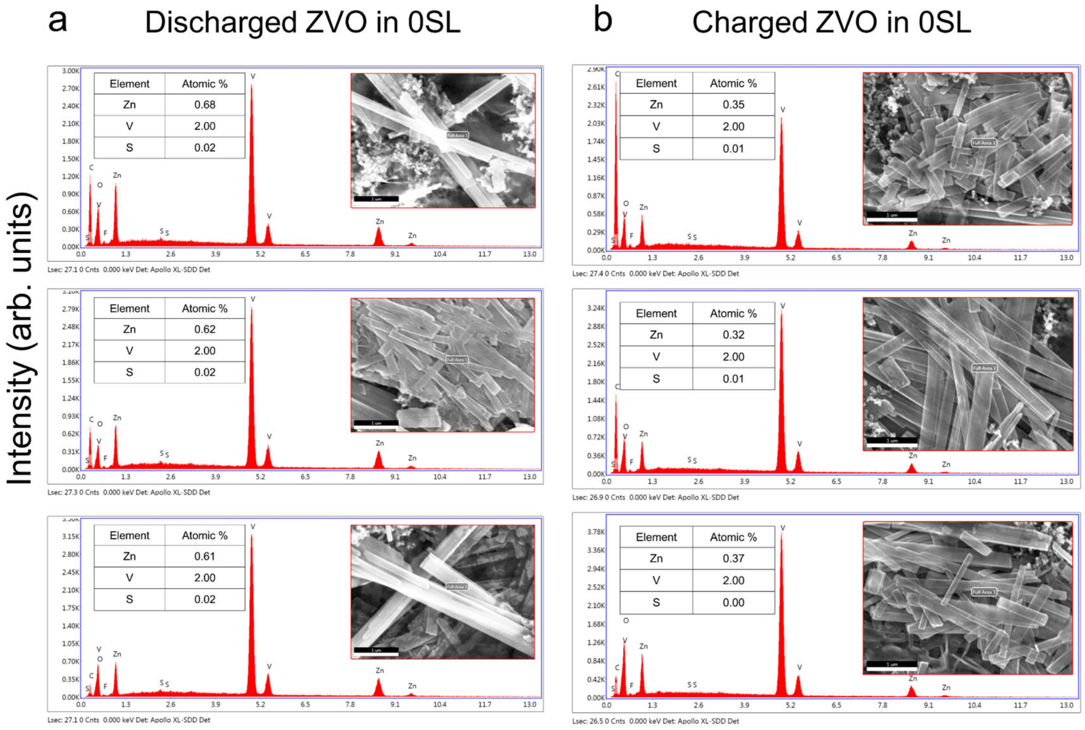

Supplementary Fig. 6 | Three representative samples of EDS data for the **(a)** discharged and **(b)** charged ZVO cathode in 0SL at 0.5C.

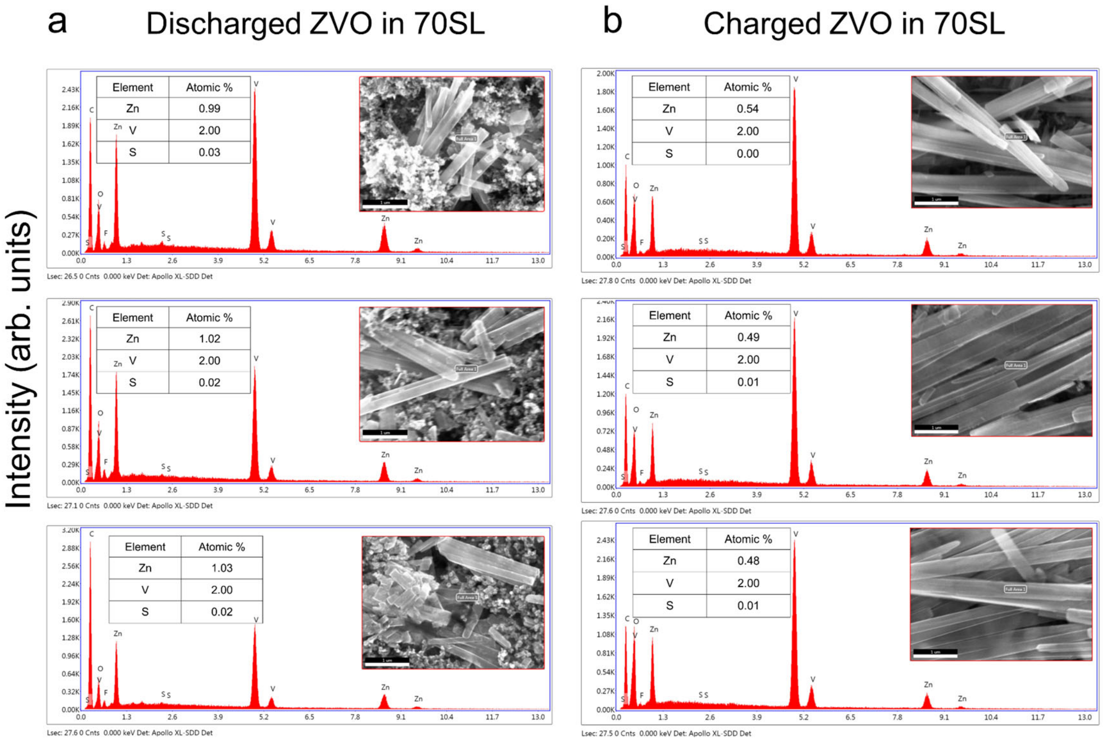

Supplementary Fig. 7 | Three representative samples of EDS data for the **(a)** discharged and **(b)** charged ZVO cathode in 70SL at 0.5C.

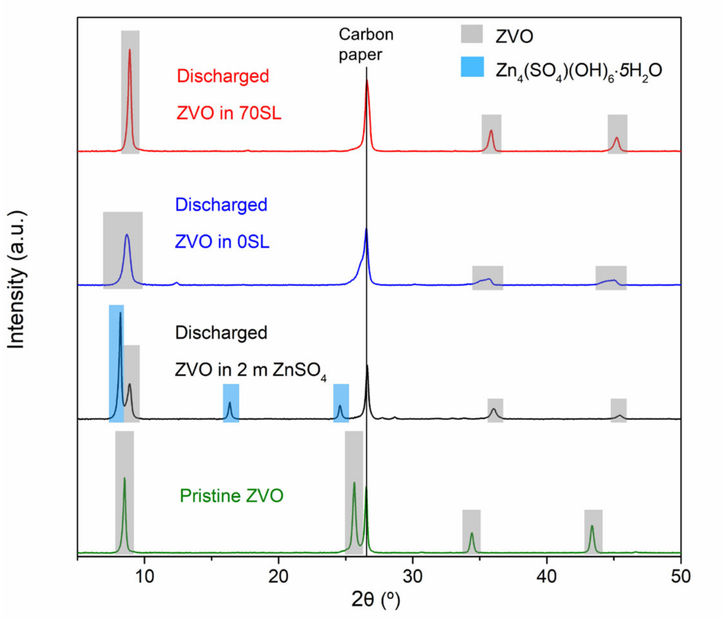

Supplementary Fig. 8 | XRD patterns of the first discharge of ZVO in different electrolytes at 0.5C. Compared to **Fig. 2e** and **2f**, for ZVO discharged in 0SL, triflate-based LDH ($Zn_{12}(CF_3SO_3)_9(OH)_{15}\cdot nH_2O$) disappeared after vigorously washing, whereas sulfate-based LDH ($Zn_4(SO_4)(OH)_6\cdot 5H_2O$) was preserved for ZVO discharged in 2 m $ZnSO_4$. This is probably due to the bulky nature of triflate anions compared to the more tightly bonded sulfate anion in LDHs.`

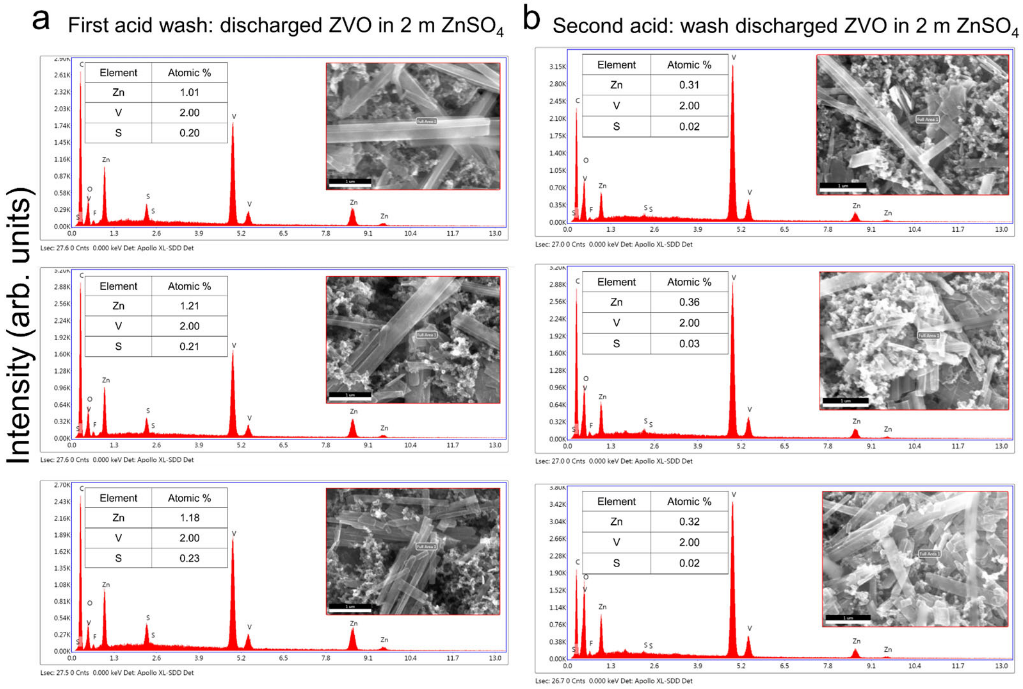

Supplementary Fig. 9 | Three representative samples of EDS data for **(a)** the first acid-washed and **(b)** the second acid-washed discharged ZVO cathode in 2 m $ZnSO_4$ at 0.5C.

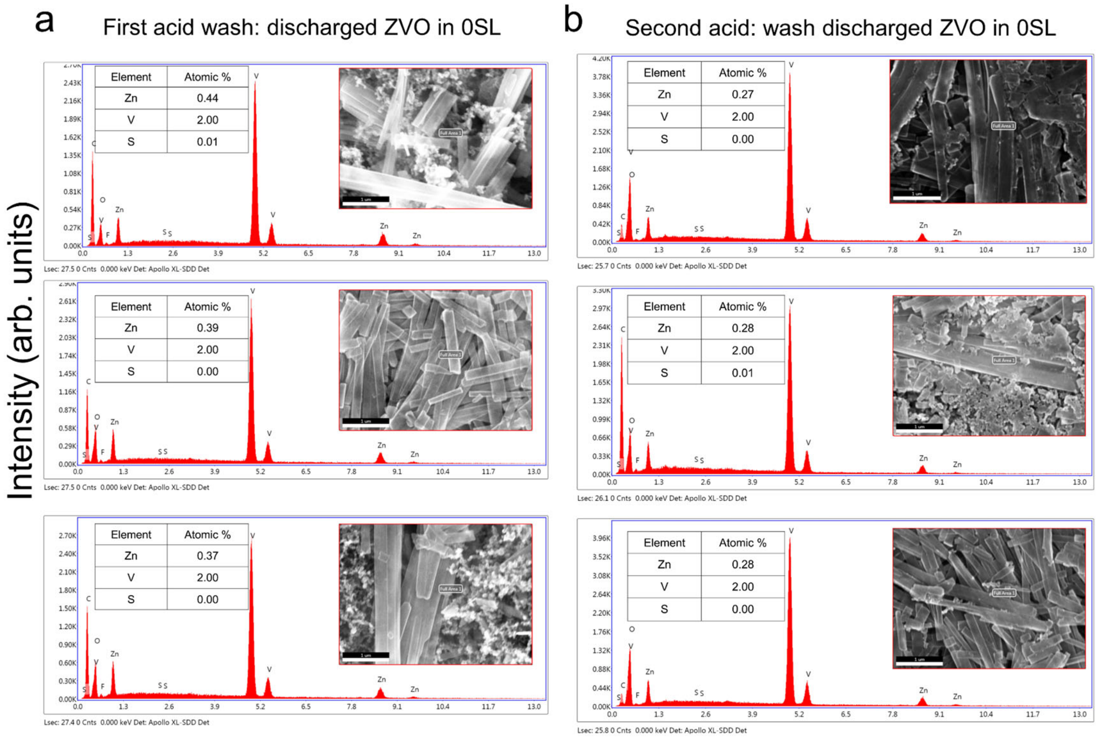

Supplementary Fig. 10 | EDS of **(a)** the first acid-washed and **(b)** the second acid-washed discharged ZVO cathode in 0SL at 0.5C.

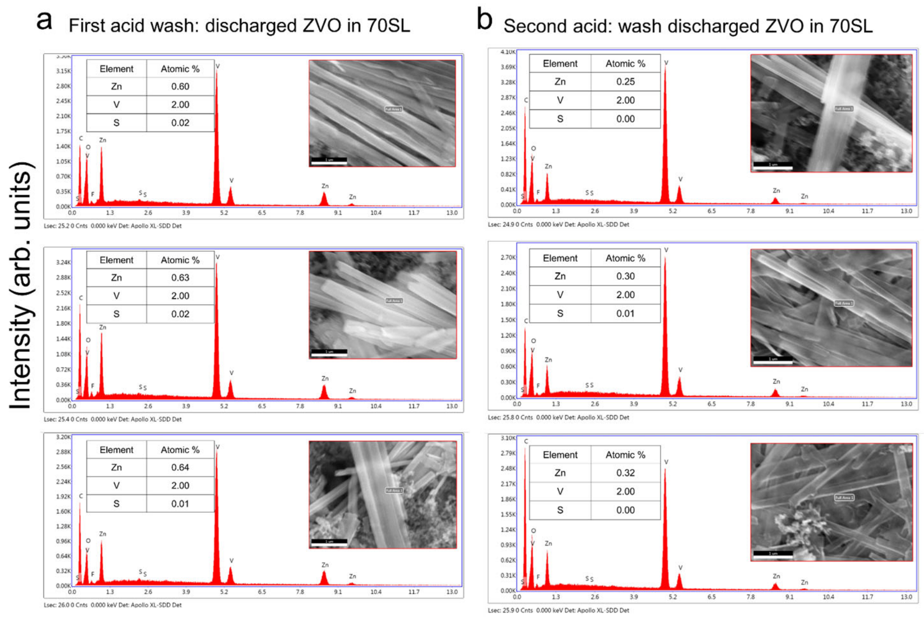

Supplementary Fig. 11 | EDS of **(a)** the first acid-washed and **(b)** the second acid-washed discharged ZVO cathode in 70SL at 0.5C.

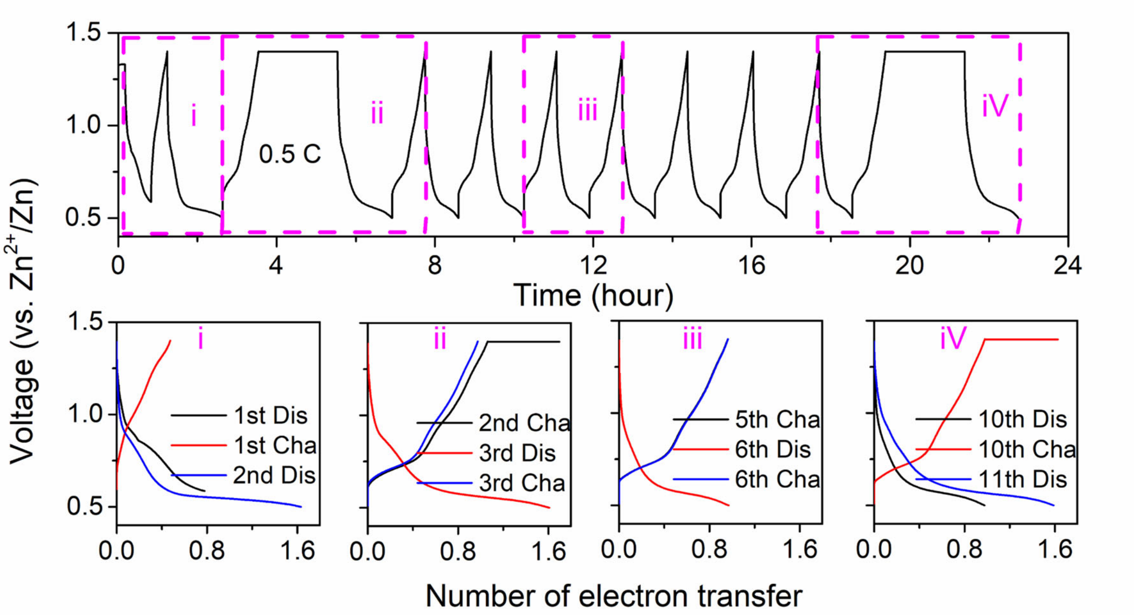

Supplementary Fig. 12 | Voltage profiles of ZVO with/without holding the potential at the fully charged state in 70SL. The cell that was initially discharged to ~ 0.6 V and achieved ~ 0.8 electron transfer (**i**), however, only ~ 0.5 electron transfer was obtained on charge. In subsequent cycles, without holding the potential at 1.4 V at the end of charge (**iii**), the electron transfer for each redox process (in the voltage range of 0.5 - 1.4 V) was only ~1.0. However, after holding the potential at the fully charged state for two hours (**ii** and **iv**), a much higher electron transfer of ~1.6 was obtained on subsequent discharge. These results indicate that Zn-ion diffusion is limited in the ZVO host, especially in the charged state.

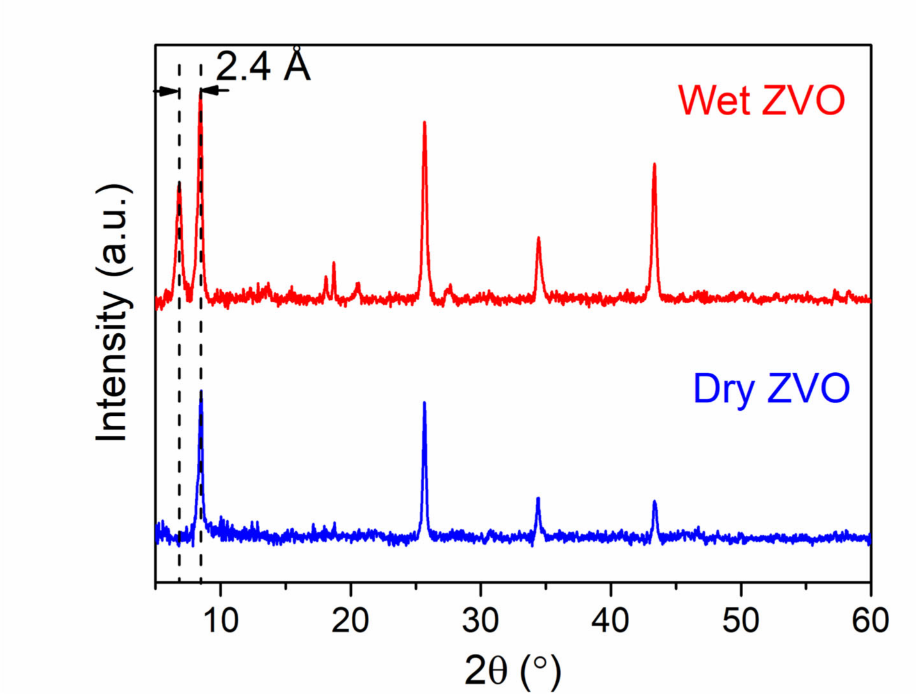

Supplementary Fig. 13 | XRD patterns of dry and wet ZVO electrodes. Upon immersion in the aqueous electrolyte, the interlayer distance increases from 10.5 Å to 12.9 Å. The difference is 2.4 Å, which is in excellent accord with values of 2-2.5 Å reported for water intercalation in other layered oxides or hydroxides.[1, 2, 3]

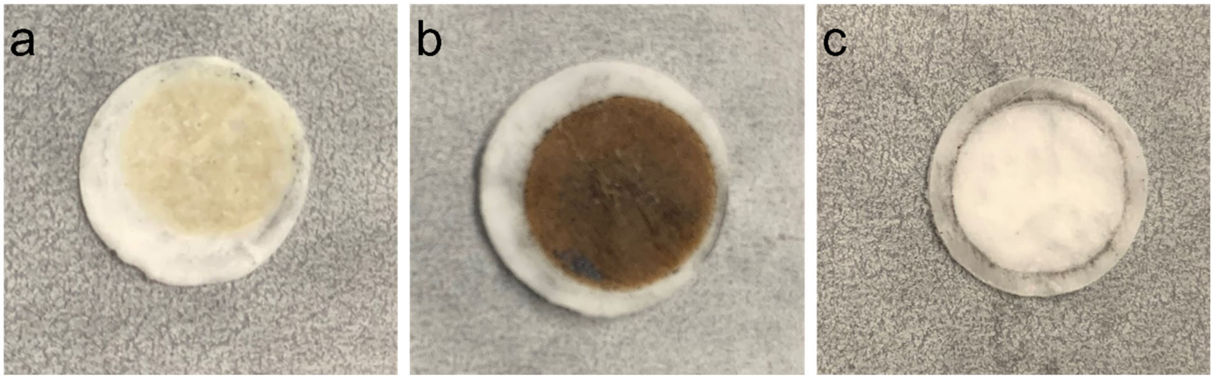

Supplementary Fig. 14 | Glass fiber separators in ZVO||Zn cells after **(a)** 120 cycles at 0.5C in 0SL and **(b)** 4000 cycles at 10C in 0SL and **(c)** 350 cycles at 0.5C in 70SL.

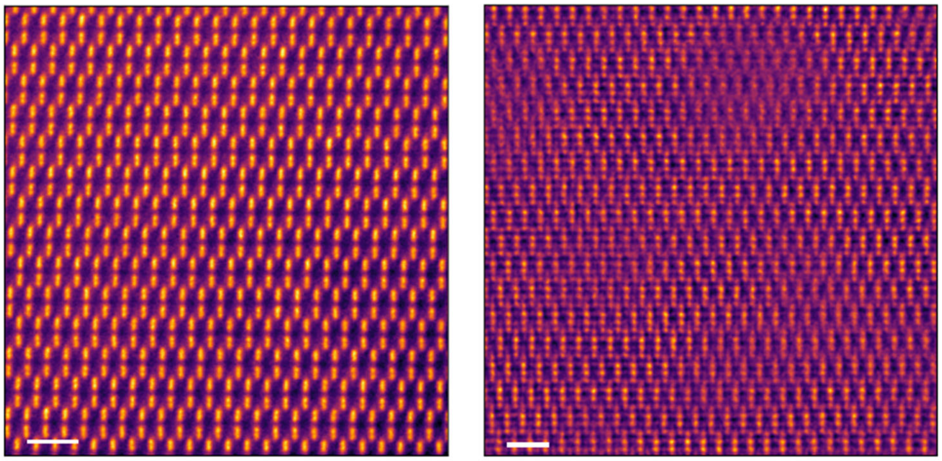

Supplementary Fig. 15 | Atomic-resolution HAADF images showing various crystal structure orientations for the pristine ZVO samples. The scale bars correspond to 1 nm.

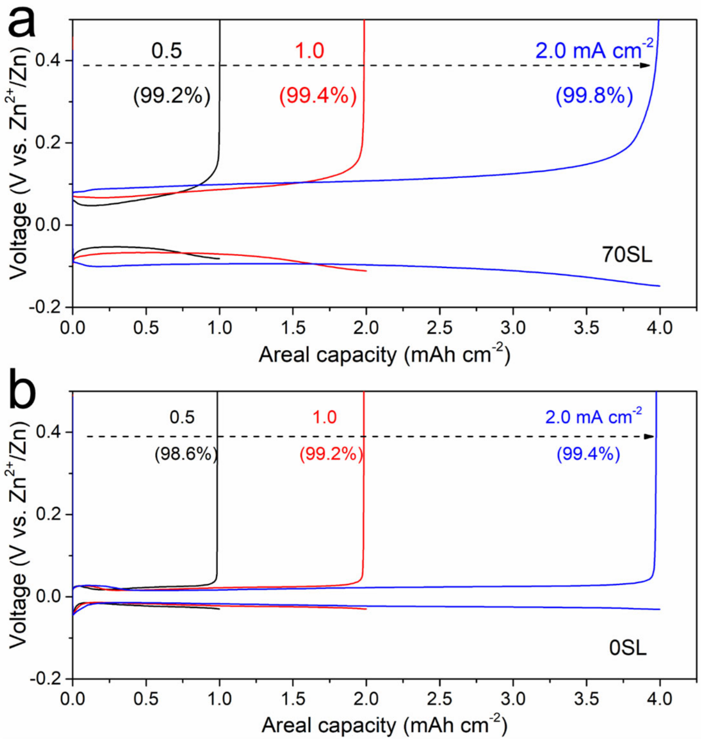

Supplementary Fig. 16 | The second voltage profiles of Cu||Zn cells at different current densities in **(a)** 70SL and **(b)** 0SL.

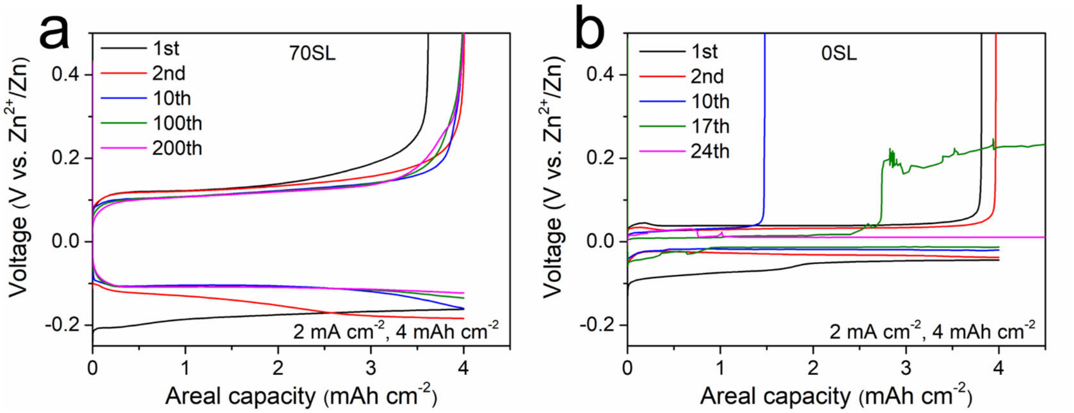

Supplementary Fig. 17 | Voltage profiles of Zn stripping/plating in Zn||Cu asymmetric cells at 2 mA cm^{-2} with a capacity of 4 mAh cm^{-2}, in **(a)** 70SL and **(b)** 0SL.

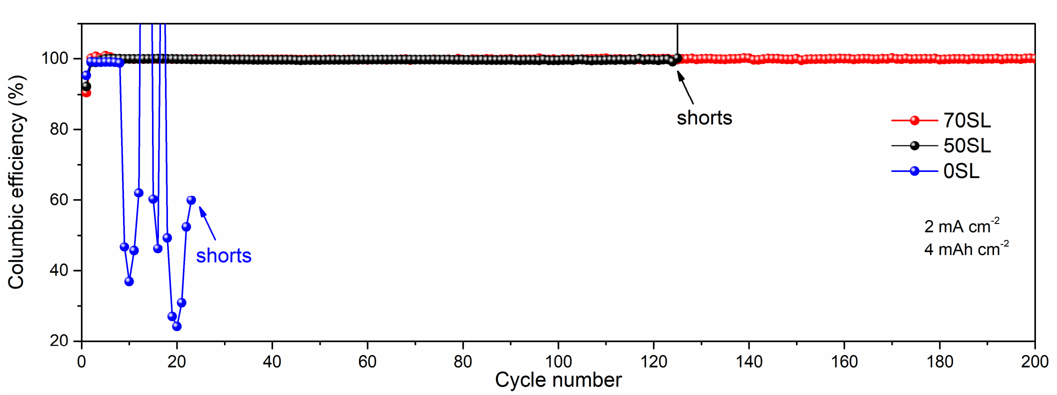

Supplementary Fig. 18 | Coulombic efficiency of Zn||Cu cells at 2 mA cm^{-2} with a capacity of 4 mAh cm^{-2} in different electrolytes.

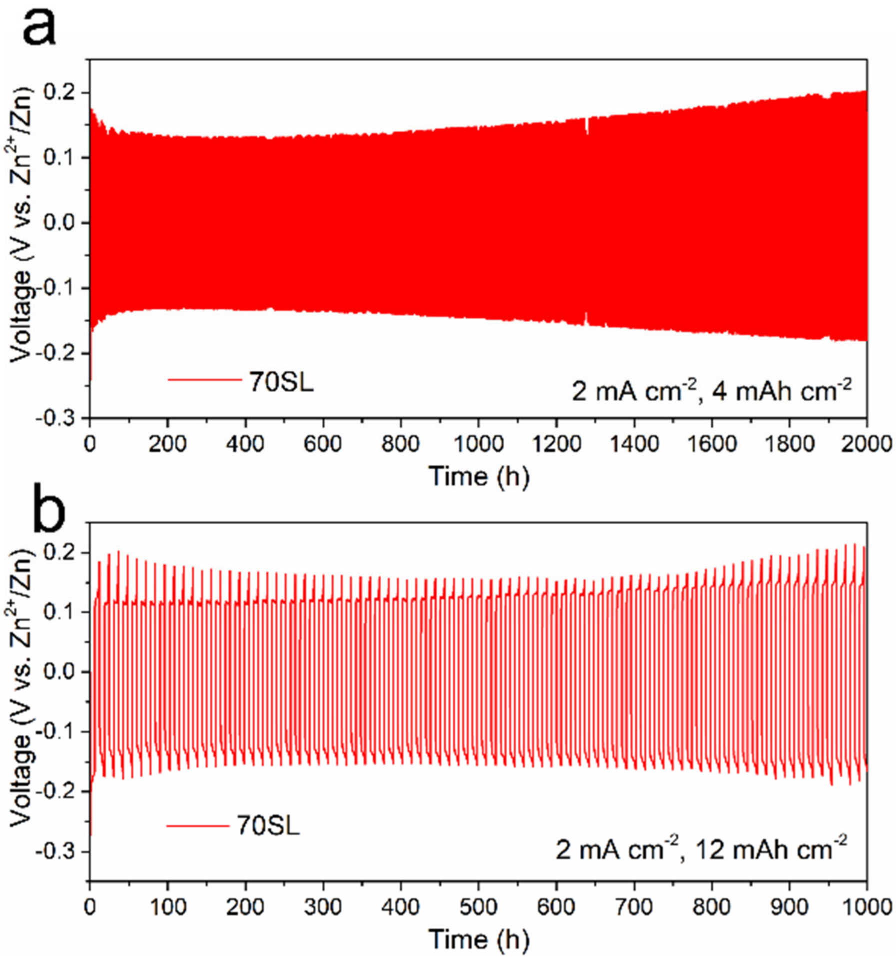

Supplementary Fig. 19 | Galvanostatic Zn stripping/plating in Zn||Zn symmetric cells with 70SL at 2 mA cm^{-2} with a capacity of (**a**) 4 mAh cm^{-2} and **(b)** 12 mAh cm^{-2}.

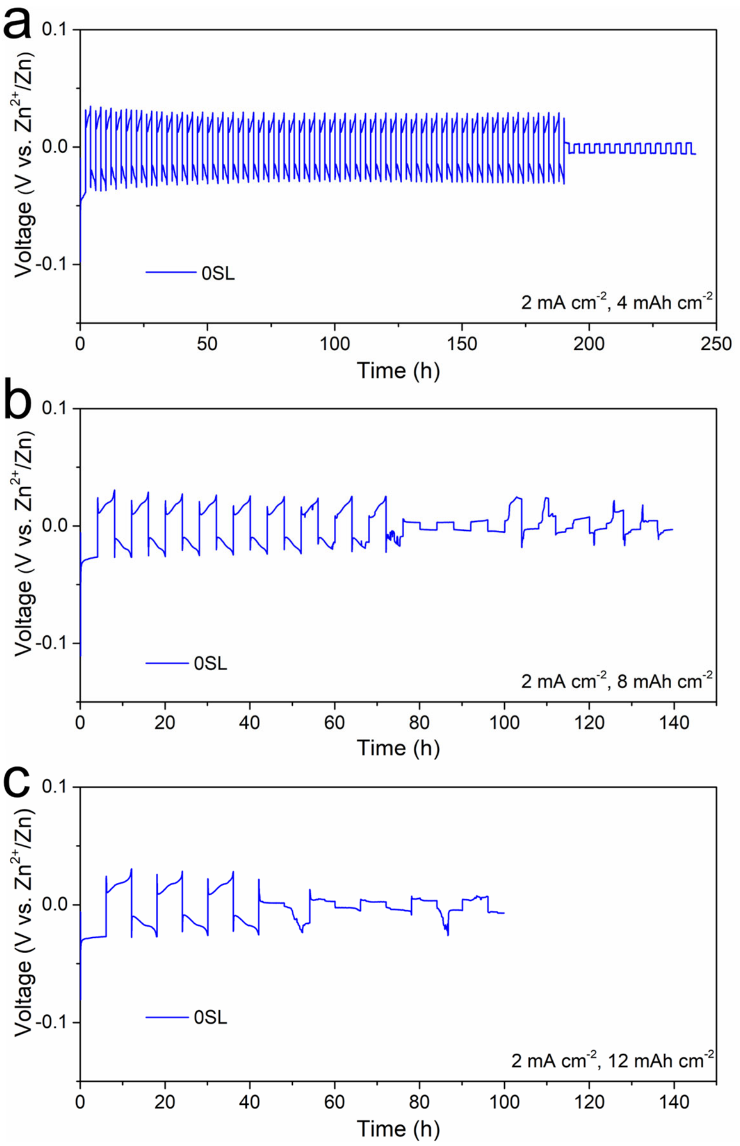

Supplementary Fig. 20 | Galvanostatic Zn stripping/plating in Zn||Zn symmetric cells with 0SL at 2 mA cm^{-2} with a capacity of (**a**) 4 mAh cm^{-2}, **(b)** 8 mAh cm^{-2} and **c)** 12 mAh cm^{-2}.

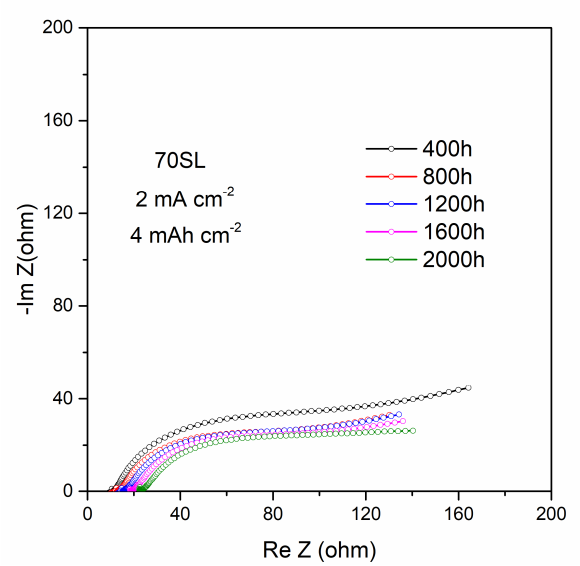

Supplementary Fig. 21 | Nyquist plots of Zn || Zn symmetric cells in 70SL after cycling at areal capacities of 2 mA cm^{-2} and 4 mAh cm^{-2} for different time periods as indicated.

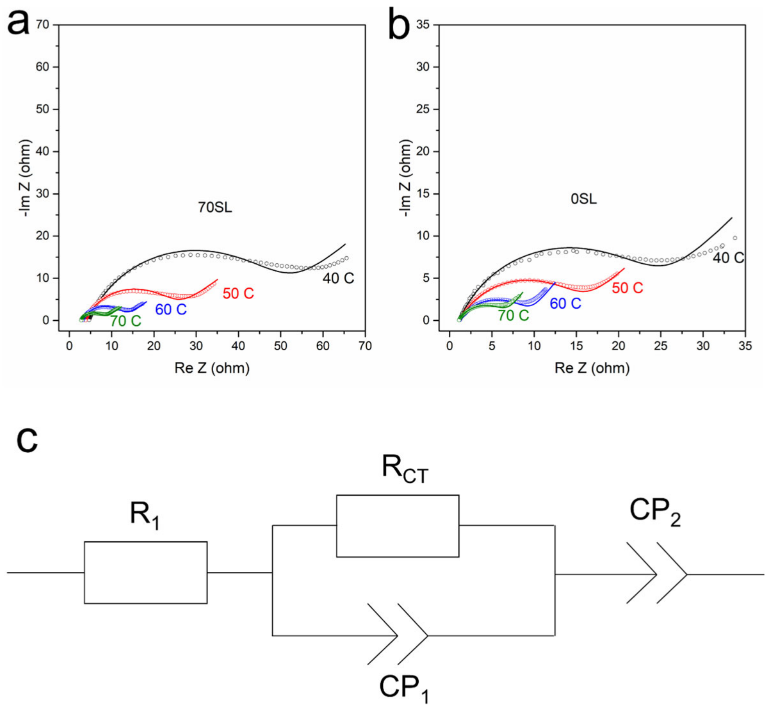

Supplementary Fig. 22 | EIS fitting of charge transfer resistance in three-electrode Zn symmetric cells in **(a)** 0SL and **(b)** 70SL at various temperatures. The data were fit (shown by the line) with **(c)** the indicated equivalent circuit.

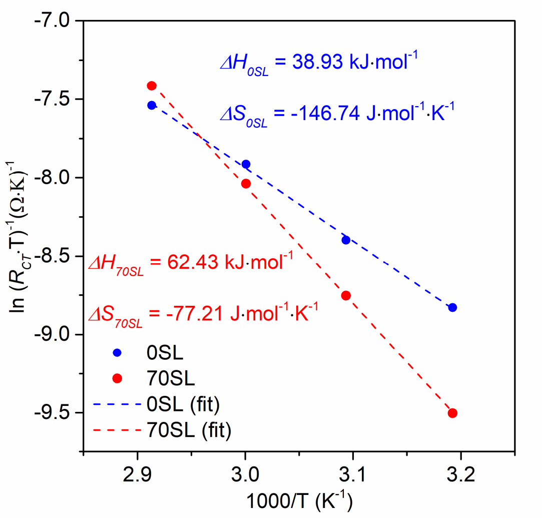

Supplementary Fig. 23 | Plots of $\ln(R_{CT}{\cdot}\mathrm{T})^{-1}$ vs. reciprocal temperature at different temperatures (from 40 °C to 70 °C) for the working electrode in a three-electrode cell with Zn as the working, counter and reference electrode. The enthalpy (ΔH) and entropy (ΔS) of interfacial charge transfer in different electrolytes were extrapolated from the slope and intercept of fitted lines based on the Eyring-Polanyi equation (S1):

$$\frac{1}{R_{CT}} = \frac{k_B T}{h} \times e^{\frac{\Delta S}{R} - \frac{\Delta H}{RT}} \tag{S1}$$

where k_{B} is Boltzmann's constant, h is Planck's constant and R is the ideal gas constant.

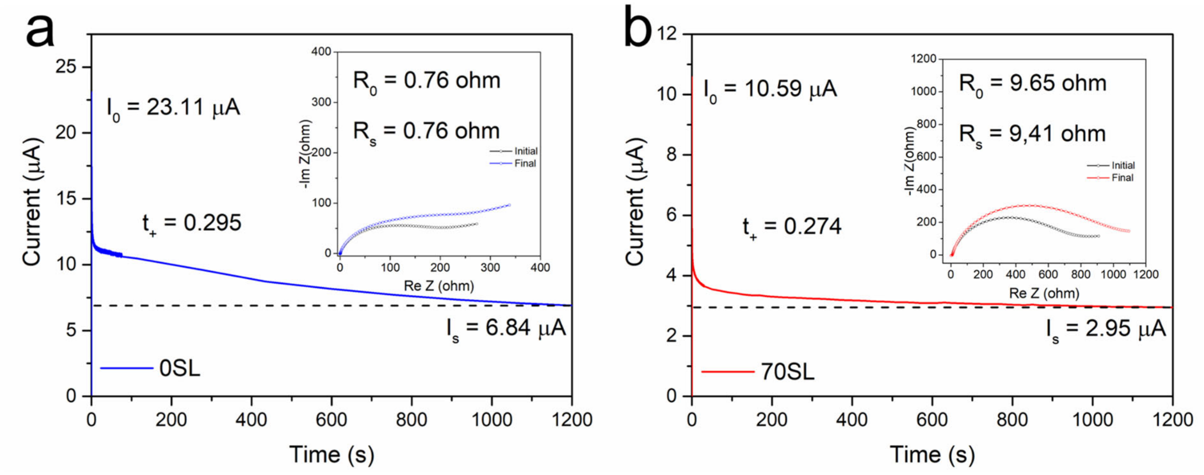

Supplementary Fig. 24 | Transference number measurements using Zn//Zn symmetric cells following a constant polarization of 5 mV for 1200 s. **a** 0SL, **b** 70SL (Insets are the corresponding initial and final EIS plots). The transference number of Zn^{2+} cation (**t_+**) was determined by equation (S2)[4, 5]:

$$t_+ = \frac{Is(\Delta V - IoRo)}{Io(\Delta V - IsRs)} \tag{S2}$$

where ΔV is the applied constant polarization voltage (5 mV), Io and Ro are the initial current and resistance, respectively; Is and Rs are the steady-state current and resistance, respectively.

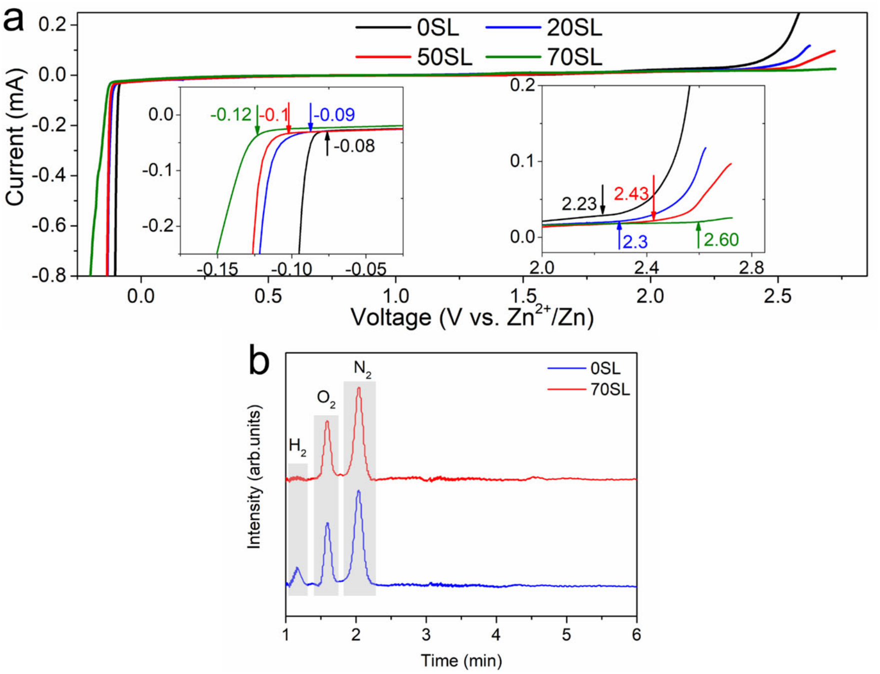

Supplementary Fig. 25 | a Linear sweep voltammetry (LSV) curves of different electrolytes. **b** Gas chromatograph response for HER in different electrolytes, which was collected after plating at 4 mA cm^{-2} for 20 min in Zn||Cu asymmetric cells.

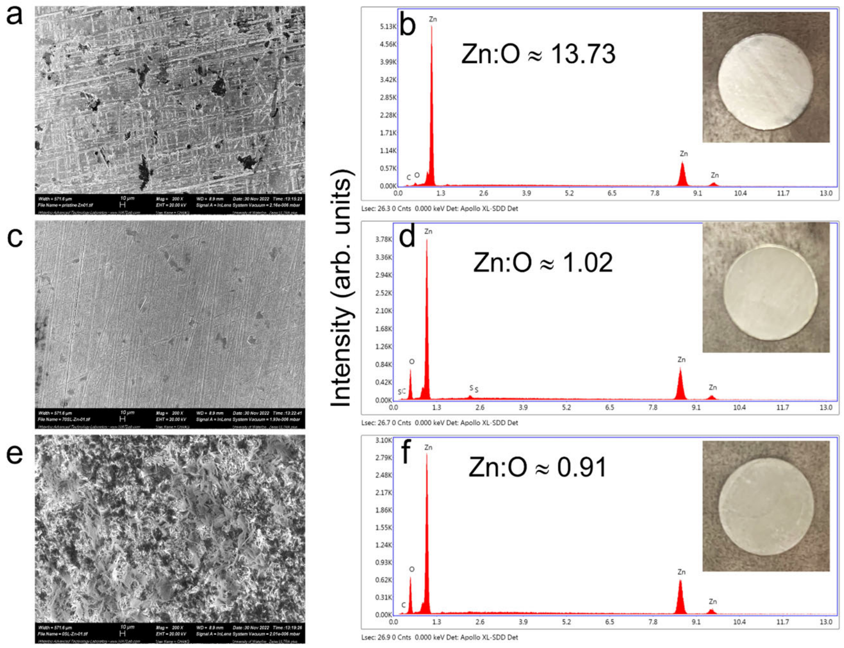

Supplementary Fig. 26 | SEM images and corresponding EDX maps of **(a, b)** pristine Zn and Zn soaked in **(c, d)** 70SL and **(e, f)** 0SL for 8 days. Although the soaked Zn in both electrolytes show the formation of ZnO on the surface, Zn in 70SL shows much smoother surface and less ZnO, indicating the better compatibility due to the much lower free water in 70SL.

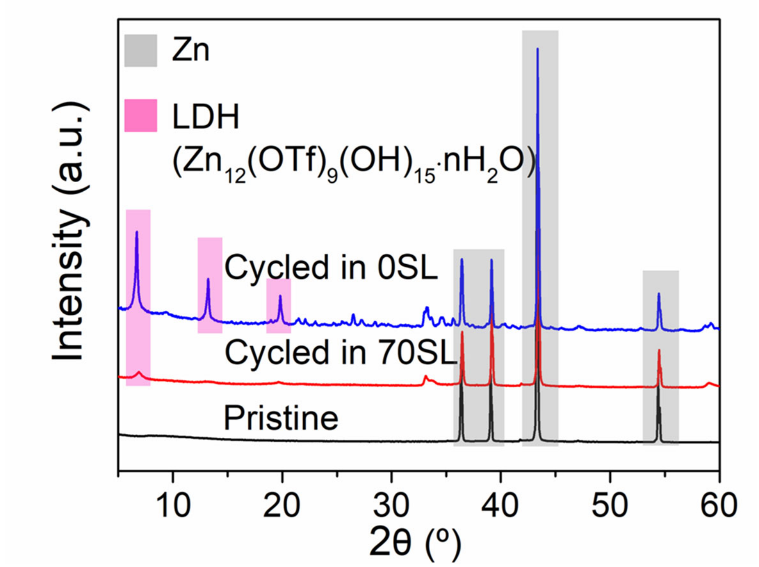

Supplementary Fig. 27 | XRD of deposited Zn on a Cu substrate after 5 cycles in different electrolytes at 2 mA cm^{-2} and 4 mAh cm^{-2} in different electrolytes.

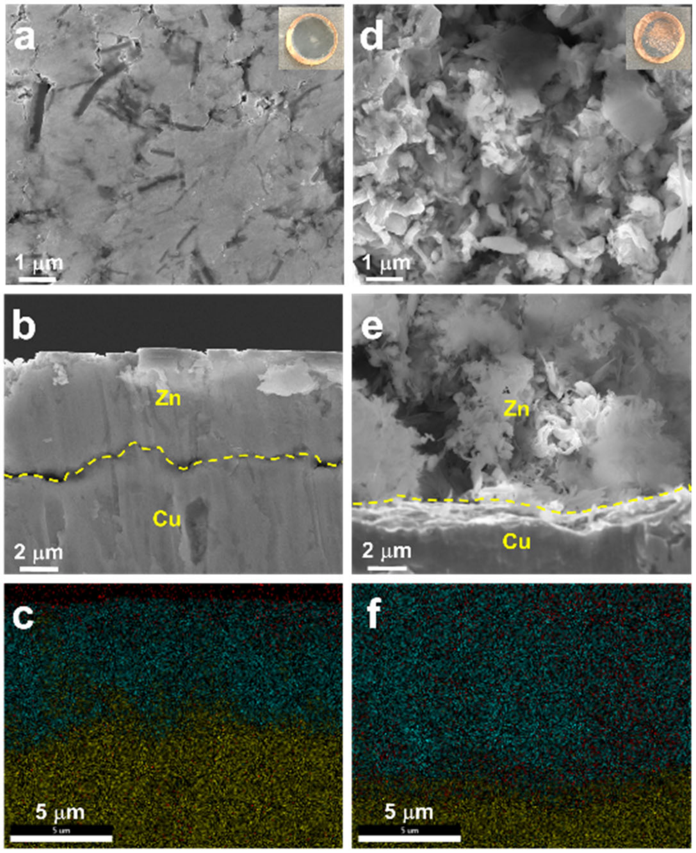

Supplementary Fig. 28 | Top and cross-sectional (**a, b, d, e**) SEM images and (**e, f**) corresponding EDX mapping of deposited Zn on Cu substrate after 5 cycles in **(a-c)** 70SL and **(d-f)** 0SL at 2 mA cm^{-2} and 4 mAh cm^{-2}.

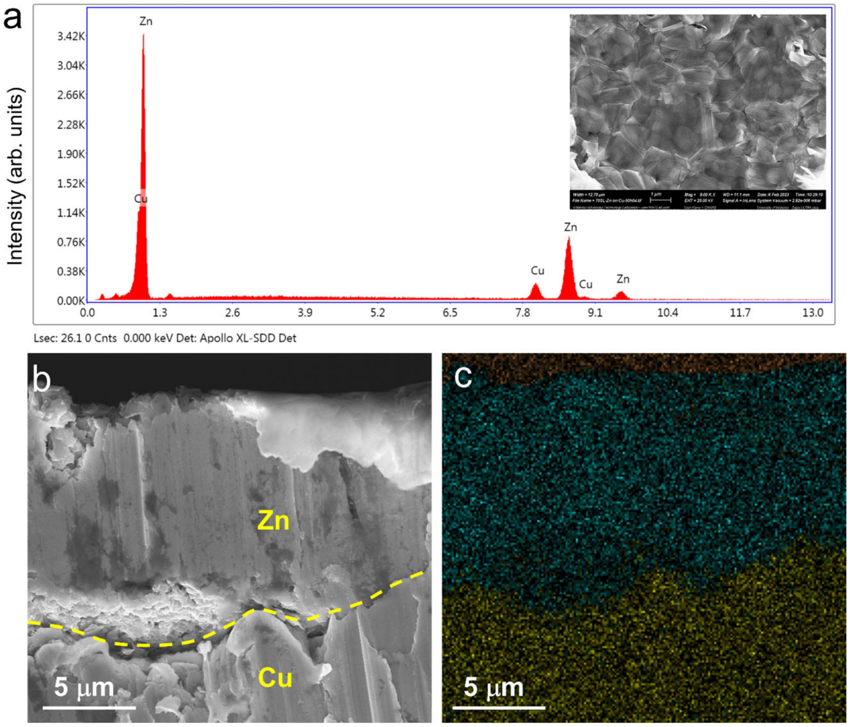

Supplementary Fig. 29 | Morphologies of 50th cycled Cu electrode with deposited Zn in 70SL at 2 mA cm^{-2} and 4 mAh cm^{-2}. (**a**) EDX and corresponding top-view image. (**b**) Cross-sectional view and (**c**) corresponding EDX mapping images. The smooth and dense morphology of plated Zn after 50 cycles confirms the dendrite-free and highly stable Zn plating/striping in 70SL.

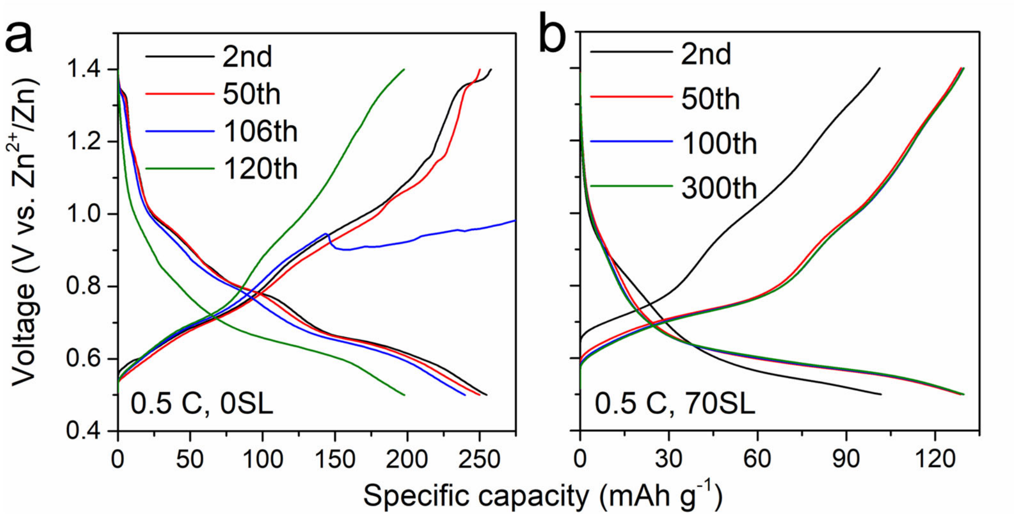

Supplementary Fig. 30 | Voltage profiles of ZVO in different electrolytes at 0.5C in **(a)** 0SL and **(b)** 70SL.

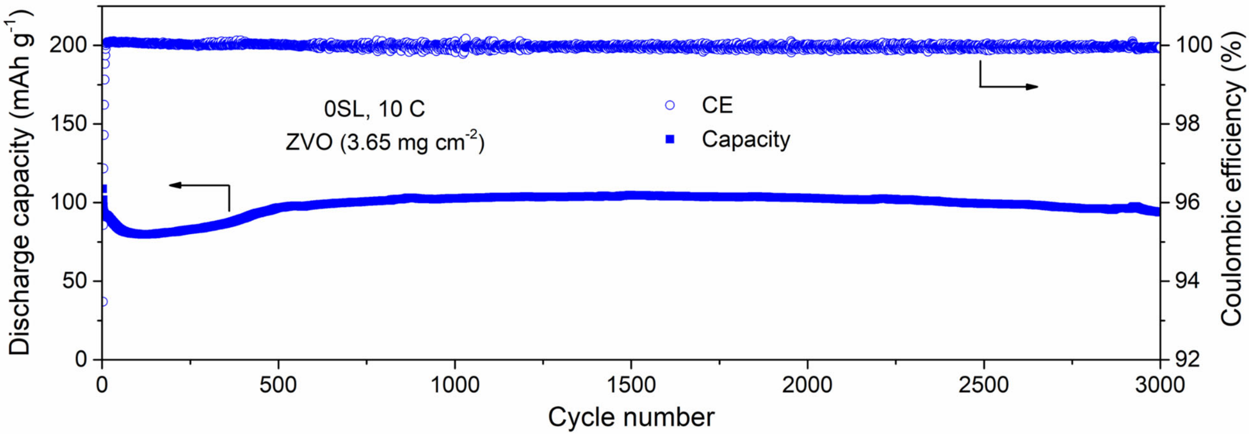

Supplementary Fig. 31 | The discharge capacity of a ZVO||Zn full cell as a function of cycle number in 0SL at 10C.

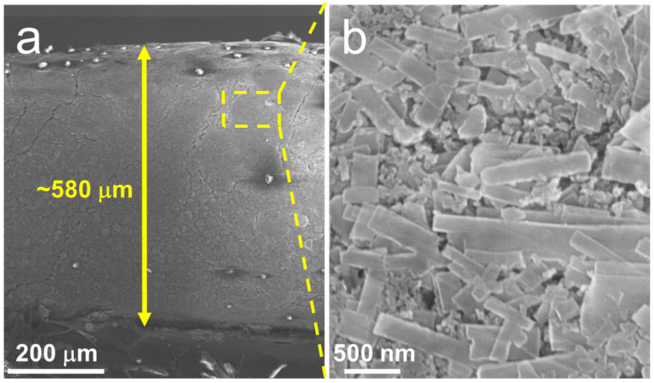

Supplementary Fig. 32 | a,b SEM images of the high-loading and freestanding ZVO cathode **(a)** and a magnified view of the indicated region **(b)**.

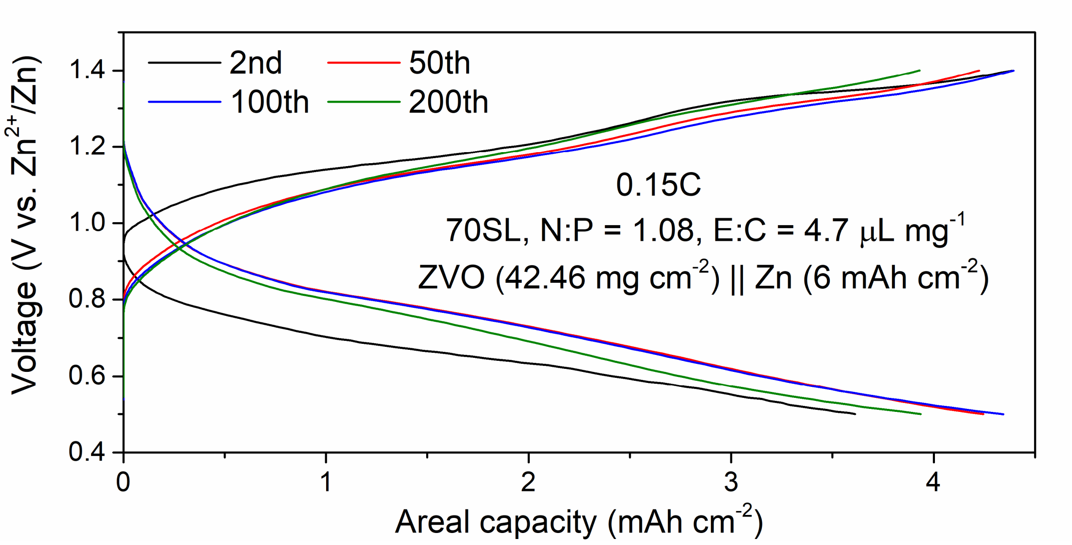

Supplementary Fig. 33 | Voltage profile of high-loading ZVO in 70SL at 0.15C with a controlled N/P ratio of 1.08.

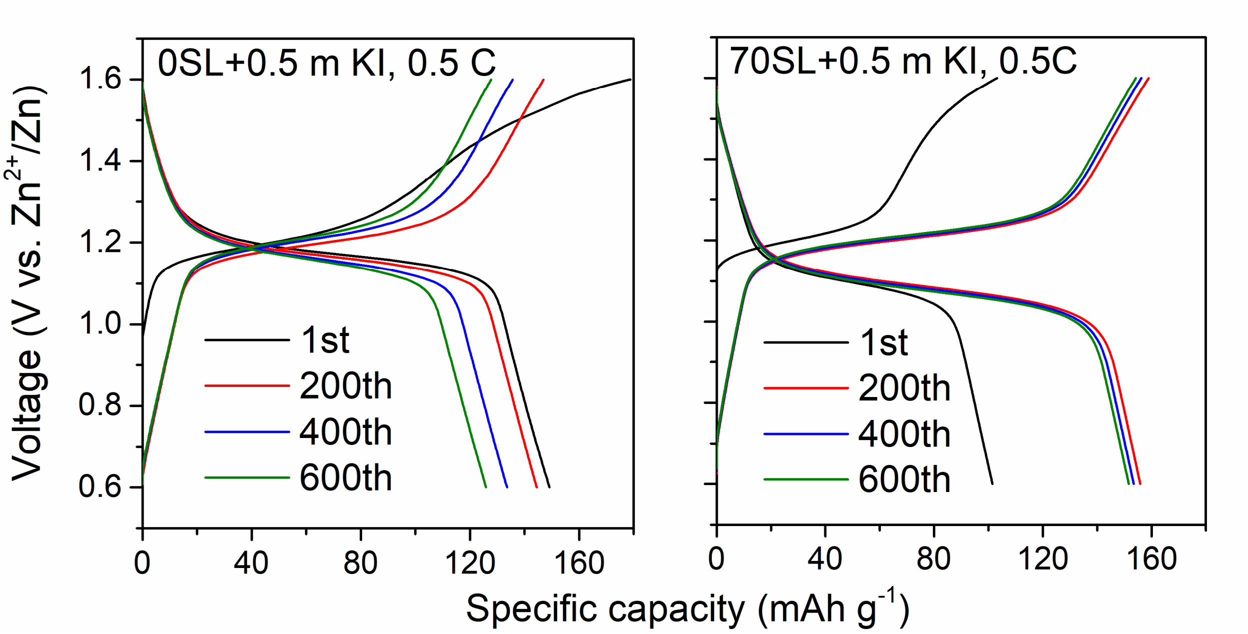

Supplementary Fig. 34 | Voltage profiles of activated carbon (AC) electrodes in different electrolytes with the addition of 0.5 m KI at 0.5C

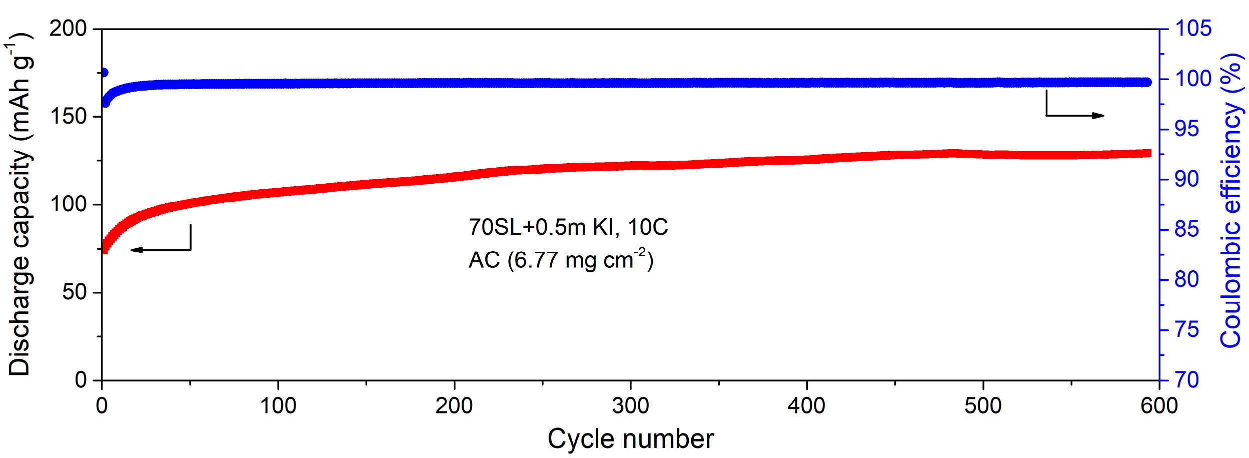

Supplementary Fig. 35 | The cycling stability of AC electrodes in 70SL with the addition of 0.5 m KI at 10C

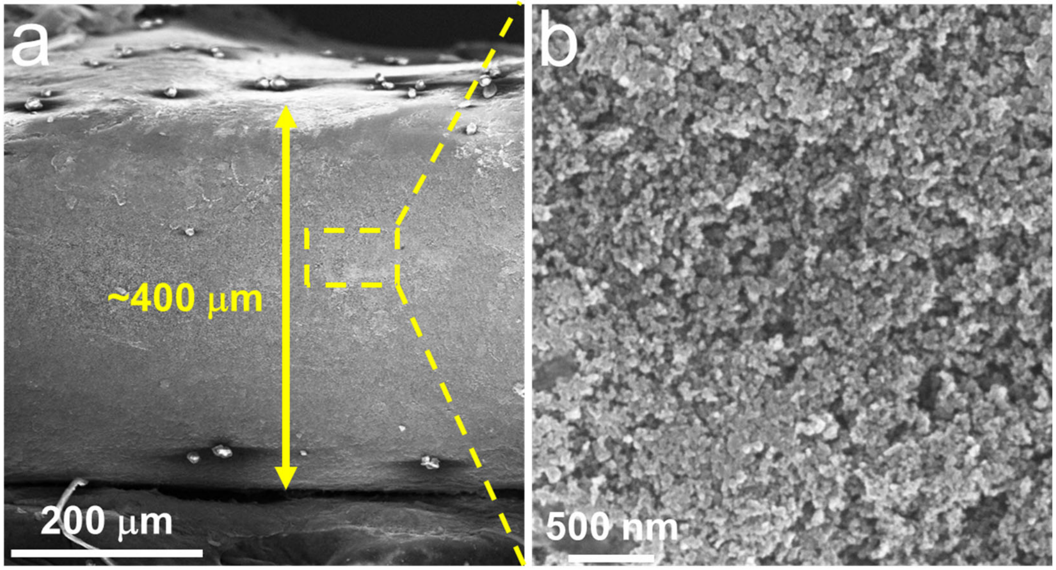

Supplementary Fig. 36 | a,b SEM images of the high-loading and freestanding activated carbon cathode **(a)** and a magnified view of the indicated region **(b)**.

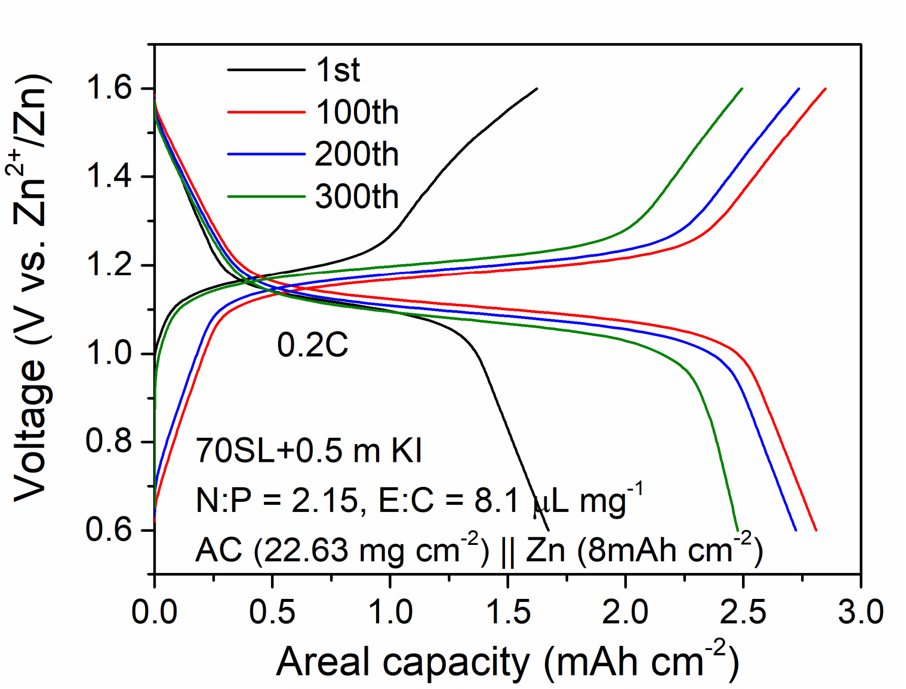

Supplementary Fig. 37 | Voltage profiles of high-loading AC in 70SL with addition of 0.5 m KI at 0.2 C at a controlled N/P ratio of 2.15.

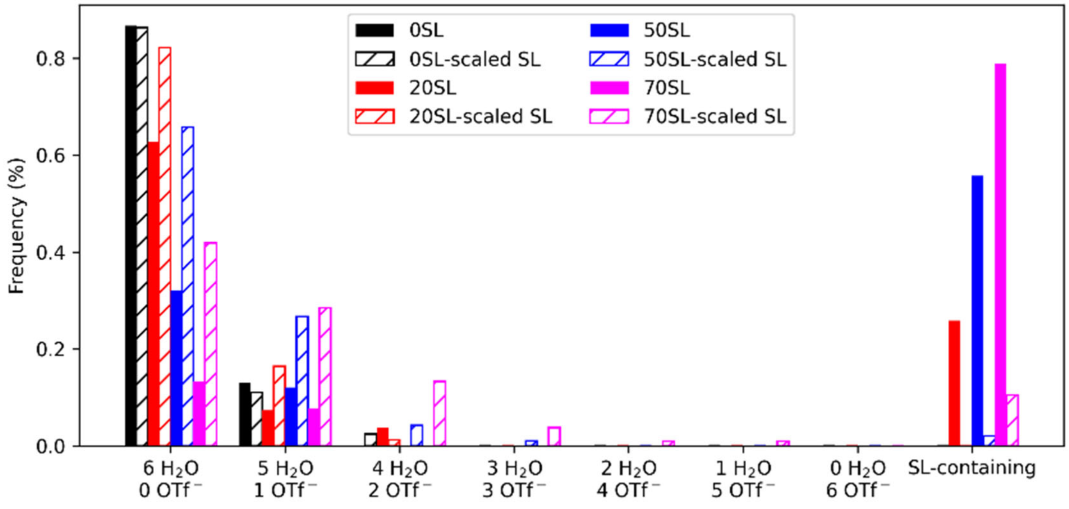

Supplementary Fig. 38 | Distribution of different solvation shell configurations from classical MD simulations with and without charge scaling on the SL molecule. Fully-hydrated Zn^{2+} corresponds to the "**6** H_2O + **0** OTf^-" category. The "SL-containing" category represents any solvation configuration containing at least 1 SL. Note that the total coordination number of Zn^{2+} was found to be 6 in virtually all cases. Charges on the Zn^{2+} and OTf^- ions were scaled by 0.8x in all simulations shown.

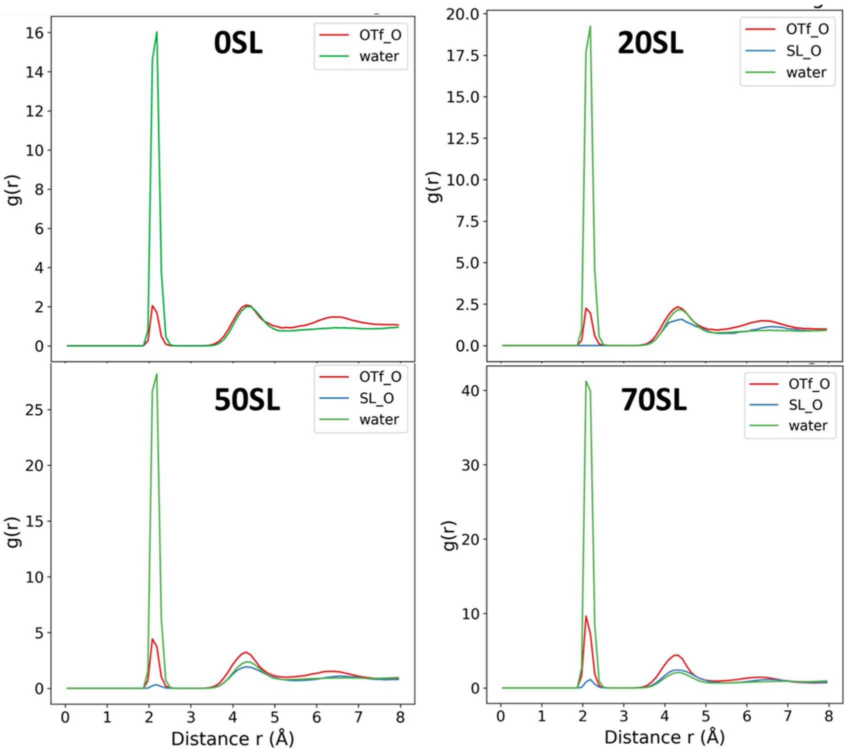

Supplementary Fig. 39 | Radial distribution functions (RDFs) of H_2O-O, OTf-O, and SL-O around Zn^{2+} in different electrolytes obtained from classical MD simulations.

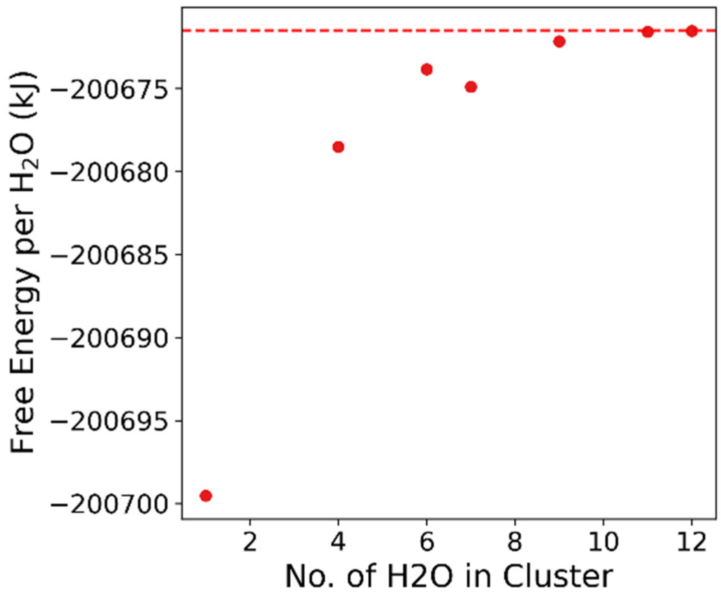

Supplementary Fig. 40 | Convergence of DFT-computed free energy of water with increasing water cluster size.

Supplementary Table 1. Summary of prices, boiling points and flash points of various nonaqueous solvents. Price data of all solvents and flash point of trimethyl phosphate were collected from Sigma Aldrich (https://www.sigmaaldrich.com/US/en), while other boiling points and flash points were collected from Wikipedia (https://en.wikipedia.org/wiki).

Solvents	Sigma Aldrich SKU	Online price (US$)	Normalized price (US$/kg)	Boiling point (°C)	Flash point (°C)
methanol	322415	93.4/L	118	64.7	11.5
sulfolane	T22209	44.3/100g	443	285	165
ethylene glycol (EG)	324558	59.8/100ml	537	197.3	111
propylene carbonate (PC)	310328	71.5/100ml	593.8	242	132
dimethyl carbonate (DMC)	517127	65.2/100ml	609	90	17
trimethyl phosphate (TMP)	241024	36.4/50g	728	197	150
succinonitrile (SCN)	160962	98/100g	980	266.1	113

Supplementary Table 2. Summary of EDS-derived elemental compositions of ZVO electrodes cycled in different electrolytes after different wash conditions. The EDX values are normalized based on $Zn_{0.25}V_2O_5$ formula with a fixed vanadium mole ratio of 2.

Sample	Elemental ratio	Normal wash (water x 5 + ethanol x 2)	1st acid wash (pH~3, HCl)	2nd acid wash (pH~3, HCl)
Discharged ZVO in 2 M $ZnSO_4$ at 0.5 C	Zn : V	1.36 : 2	1.13 : 2	0.33 : 2
	S : V	0.24 : 2	0.21 : 2	0.02 : 2
Discharged ZVO in 0SL at 0.5 C	Zn : V	0.64 : 2	0.4 : 2	0.28 : 2
	S : V	0.02 : 2	0 : 2	0 : 2
Discharged ZVO in 70SL at 0.5 C	Zn : V	1.01 : 2	0.62 : 2	0.29 : 2
	S : V	0.02 : 2	0.01 : 2	0 : 2

References

1. Buchholz, D., Chagas, L. G., Vaalma, C., Wu, L. & Passerini, S. Water sensitivity of layered P2/P3-$Na_xNi_{0.22}Co_{0.11}Mn_{0.66}O_2$ cathode material. *J. Mater. Chem. A* **2**, 13415–13421 (2014).
2. Lin, C., Chen, D., Lemmens, P., Zhang, X., Maljuk, A. & Zhang, P. Study of intercalation/deintercalation of Na_xCoO_2 single crystals. *J. Cryst. Growth* **275**, 606–616 (2005).
3. Radha, S., Jayanthi, K., Breu, J. & Kamath, P. V. Relative humidity induced reversible hydration of sulfate intercalated layered double hydroxide. *Clays Clay Miner.* **62**, 53–61 (2014).
4. Cao, L. et al. Fluorinated Interphase Enables Reversible Aqueous Zinc Battery Chemistries. *Nat. Nanotechno.* **16**, 902-910 (2021).
5. Han, D. et al. A Non-Flammable Hydrous Organic Electrolyte for Sustainable Zinc Batteries. *Nat. Sustain.* **5**, 205-213 (2021).
