# Supplementary material for: Enabling selective zinc-ion intercalation by a eutectic electrolyte for practical anodeless zinc batteries
Source: Nat Commun. 2023 May 27;14:3067. doi: 10.1038/s41467-023-38460-2 (PMC10224959; doi:10.1038/s41467-023-38460-2)
Supplement: Supplementary file 3 — Description to Additional Supplementary Information [file 41467_2023_38460_MOESM3_ESM.pdf]

Description of Additional Supplementary Files

Supplementary Movie 1. Flammability tests of glass fiber separators soaked with electrolytes.

Supplementary Movie 2. Flammability tests of electrolytes.
